# Dipolar Order Parameters in Large Systems With Fast Spinning

**DOI:** 10.3389/fmolb.2021.791026

**Published:** 2021-12-09

**Authors:** W. Trent Franks, Ben P. Tatman, Jonah Trenouth, Józef R. Lewandowski

**Affiliations:** ^1^ Department of Physics, University of Warwick, Coventry, United Kingdom; ^2^ Department of Chemistry, University of Warwick, Coventry, United Kingdom

**Keywords:** symmetry, dynamics, fast MAS NMR, recoupling, order parameter, proton detection

## Abstract

Order parameters are a useful tool for quantifying amplitudes of molecular motions. Here we measure dipolar order parameters by recoupling heteronuclear dipole-dipole couplings under fast spinning. We apply symmetry based recoupling methods to samples spinning under magic angle at 60 kHz by employing a variable flip angle compound inversion pulse. We validate the methods by measuring site-specific ^15^N-^1^H order parameters of a microcrystalline protein over a small temperature range and the same protein in a large, precipitated complex with antibody. The measurements of the order parameters in the complex are consistent with the observed protein undergoing overall motion within the assembly.

## Introduction

The astounding number of structures found in the protein databank speaks to the usefulness of structural data to provide insights into the structure-function relationship in biology and biochemistry (Berman et al., 2000; Burley et al., 2021). With advent of powerful computational structure prediction approaches such as AlphaFold (Jumper et al., 2021) and RoseTTAFold (Baek et al., 2021) there is an almost exponential increase of systems for which a reasonable quality structure or models become available. However, a structure is a snapshot that does not necessarily capture the choreography of the protein it needs to execute in order to perform its function (Koshland, 1958; Frauenfelder et al., 1991). The motion of a protein is often intrinsic to its activity. Understanding the dynamics and how structure changes in time is sometimes nearly as important as knowing a single, even high-resolution, snapshot. An ultimate example of this idea are intrinsically disordered proteins (IDP) and intrinsically disordered regions (IDR), which are involved in controlling countless processes in eukaryotic but also prokaryotic organisms, *e.g.* biosynthetic steps in production of bioactive natural products (Jenner et al., 2018; Kosol et al., 2019; Fage et al., 2021).

Nuclear magnetic resonance (NMR), can be used to find molecular motions under near physiological conditions at atomic resolution over a several orders of magnitude of the time scale, from as fast as picoseconds, to as slow as months, but will only report on local conditions and typically over small distance scales (Palmer, 2004; Kovermann et al., 2016; Sekhar and Kay, 2019; Alderson and Kay, 2021). However, to access such a vast range of dynamics a battery of different tools reporting on different parameters of motion in different regimes is required. For example, NMR relaxation is sensitive to both amplitudes and time scales of motions typically in the picoseconds-nanoseconds range in solution and picoseconds-milliseconds range in the solid state (Lewandowski, 2013), which provides some unique opportunities for characterizing protein motions (Castellani et al., 2002; Chevelkov et al., 2003; Chevelkov et al., 2006; Lewandowski et al., 2011; Asami and Reif, 2012; Lamley et al., 2014; Lamley et al., 2015a; Lamley et al., 2015b; Sternberg et al., 2018; Öster et al., 2019). However, the extended range of time scales of motions, which influence relaxation in the solid state, comes also at a price: reliable quantification of motional amplitudes with relaxation rates alone is challenging and sometimes impossible. Often reliable quantification of dynamics using relaxation rates requires them to be combined with measurements of order parameters (typically dipolar order parameters), which constrain the overall amplitude of motions (Schanda and Ernst, 2016).

Order parameters can be obtained by recoupling specific terms of the NMR Hamiltonian in a separated local field (SLF) experiment (Hester et al., 1976). SLF techniques use pulses to create a Hamiltonian where one term of the full Hamiltonian is recoupled, and the other terms are averaged to zero. Examples of such methods include C7 (Hohwy et al., 1998), RFDR (Bennett et al., 1992), REDOR (Gullion and Schaefer, 1989a; Gullion and Schaefer, 1989b), TMREV (Hohwy et al., 2000; Franks et al., 2005; Franks et al., 2006a), and many others. The recoupling portion of SLF experiments have been summarized into a uniform theory using symmetry principles (Levitt, 2002). The development has mainly been focused on slowly spinning samples, as fast rotation was not available at the time. However, fast magic angle spinning (MAS) NMR experiments, introduced since the main formulation of this theory, have improved the process of assignment and structure calculation of large proteins and complexes that were very difficult to solve using solid-state NMR otherwise (Zhou et al., 2007a; Barbet-Massin et al., 2010; Knight et al., 2012; Barbet-Massin et al., 2014). For example, the membrane protein OMPG had been actively studied for almost 15 years using carbon detection but the assignment and structure were finally solved with the use of fast spinning and proton detection (Hiller et al., 2005; Retel et al., 2017). Thus, it is desirable to extend symmetry methods to this attractive new regime. Unfortunately, symmetry sequences require applied fields that scale linearly with the spinning rate, and thus the applied field requirements cannot be usually achieved under fast spinning conditions. The application of symmetry principles to heteronuclear dipole-dipole recoupling was previously demonstrated under 40 kHz spinning (Hou et al., 2011), but did not engender optimism for application at higher spinning rates. However, spinning rates of ∼60 kHz are routine at the time of writing of this manuscript, with 100 kHz spinning becoming more common, and current cutting-edge probes reach rates on the order of 150 kHz (Penzel et al., 2019; Schledorn et al., 2020) and even 200 kHz. Consequently, symmetry methods have not been applied extensively to fast spinning samples (Brinkmann and Levitt, 2001; Levitt, 2002). SLF experiments undertaken with spinning frequencies of 60 kHz or greater have been cross-polarization-based (CP) (Chevelkov et al., 2009; Chevelkov et al., 2010; Paluch et al., 2013; Paluch et al., 2018) or use phase modulated rotary resonance pulses (Liang et al., 2021). Symmetry-based recoupling is comparable to CP based SLF experiments and has many of the same disadvantages, but symmetry be advantageous in a few ways. First, the symmetry sequences can be constructed to be very selective of the terms allowed, where only a few terms in the NMR Hamiltonian are still active. Second, there is only one channel that has high-power pulses applied which limits the power deposition, where the CP methods apply high fields on both channels simultaneously.

In this work, we introduce an approach to generate pulse sequences with optimized recoupling at fast spinning given probe performance requirements. Candidate symmetry sequences are generated, the scaling factor (κ) is optimized *in silico* using variable flip angle pulse sequence elements, and then the highest performing sequences are selected. The most promising sequences are tested against *B*_1_ inhomogeneity and match condition mis-set. The experimental performance of several candidate sequences is evaluated on a favorable model sample, the micro-crystalline protein GB1 (β1 immunoglobulin binding domain of protein G) which is uniformly labelled with ^2^H, ^13^C, ^15^N, and then back-exchanged with ^1^H at all exchangeable sites, and on a more challenging >300 kDa precipitated complex of GB1 with immunoglobulin G (IgG). We have previously investigated differences in protein dynamics for protein GB1 in these two environments. The analysis of various relaxation and relaxation dispersion experiments has indicated that while ps-ns motions and some µs motions appear to be largely similar for GB1 in the two environments, there appears to be an additional overall motional mode present only in GB1 in the complex with IgG (Lamley et al., 2015a; Öster et al., 2019). Thus we decided to investigate whether we see the presence of this additional dynamic mode reflected in the measured order parameters. In addition, we have previously performed variable temperature dynamics measurements on crystalline GB1 and in the analysis assumed the observed trends are dominated by changes to the time scales of the motions rather than changes in amplitude and assumed a constant order parameter (Lewandowski et al., 2015; Busi et al., 2018). The initial variable temperature molecular dynamics simulations suggested that indeed the ^15^N-^1^H order parameter changes only very slightly with temperature in the explored 30 °C range but we thought this study to be a good opportunity to begin to explore validity of such approximation experimentally. Incidentally, crystalline GB1 and GB1 in the complex with IgG cover the range of a favorable model sample and a challenging “real” sample.

### Symmetry Based Pulse Sequences

Symmetry-based sequences allow for the selection of portions of the full NMR Hamiltonian (Levitt, 2002). The performance of the pulse sequence with regards to the extent of the reintroduction for an interaction is indicated by a scaling factor (κ). The scaling factor is the magnitude of any coupling when compared to the static limit, which can vary between 0 and 1, where a larger κ indicates a more efficient recoupling/reintroduction. The recoupling performance can be altered in two ways: by using a different symmetry, or by using a different rotation element. This work demonstrates a strategy to find high performance heteronuclear recoupling pulse sequences by exploring the possible variations of symmetry derived pulse sequences.

The primary limitation for the application of symmetry at high spinning frequencies is the electronic performance of the probe. The nutation of the spins which are induced by the applied radio frequency field in the probe must match the conditions specified by the symmetry sequence, which is dependent on the spinning rate. The same symmetry will require higher radio frequency fields with higher spinning rates. For example, in the well-known R18_1_^7^ sequence (Levitt, 2002; Wylie and Rienstra, 2008; Wylie et al., 2011) the match condition is 9 times the spinning rate, which means that ν_1_ = 90 kHz at 10 kHz magic angle spinning (MAS) rate, but ν_1_ = 540 kHz at 60 kHz spinning frequency. The electronics in the probe will likely break down due to the high voltages needed to generate such strong pulses, or alternatively, the protein sample will denature when the temperature gets too high from radiative heating. Most modern probes are specified to work with an applied ^1^H field of ∼100 kHz for long pulses (i.e., decoupling during acquisition), with fields on the order of 250 kHz available for short periods (and nonconductive samples). Since the prior art does not produce many appropriate choices, a method to identify and optimize symmetry-based recoupling to measure the heteronuclear dipole-dipole coupling was devised.

**FIGURE 1:**
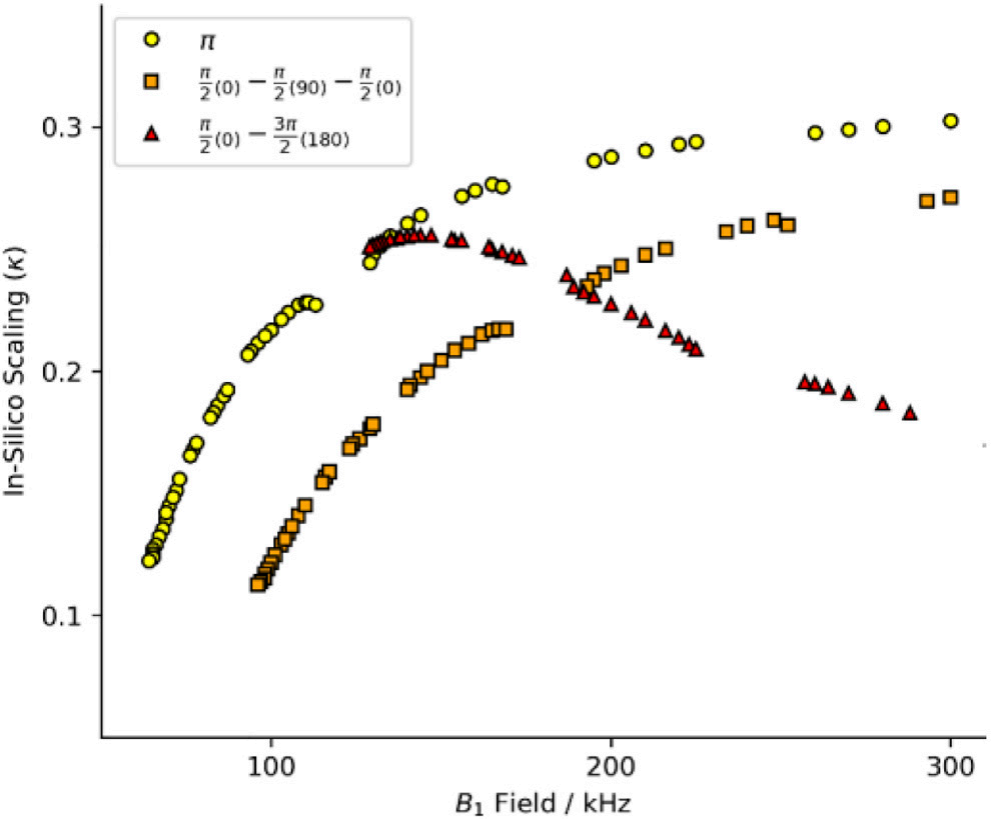
Heteronuclear dipole-dipole recoupling scaling factor for symmetries found in Hou et al. (2011). The full range of symmetry match conditions for standard composite pulses. Yellow circles are for *R*-symmetries with a standard rectangular π pulse. Red triangles report the scaling with a compound *R*-element of a π/2_(0)_−π/2_(90)_−π/2_(0)_ with the equivalent match of a 3π/2 pulse. Orange squares report the scaling with a compound *R*-element of a π/2_(0)_−3π/2_(180)_ with the equivalent match of a 2π pulse. See Supplementary Figure S1 for full *B*_1_ range.

Initially, we intended to apply a series of symmetries identified for use at 40 kHz MAS (Hou et al., 2011). We simulated these schemes at 60 kHz MAS with a standard π pulse as the R-element (see Figure 1; Supplementary Figure S1, yellow circles). The curve of scaling factor against match condition matches the literature well but the scaling factor is smaller at 60 kHz spinning than at 40 kHz (Hou et al., 2011). That is, the scaling increases with increasing field, approaching some upper limit (here, κ ∼ 0.3). We chose the best candidate symmetries to test experimentally where the criterion for selection was an applied field ν_1_ < 130 kHz. After careful calibration of the applied field using long nutation experiments, the performance of the candidate sequences was found to be poor, with little to no recoupling apparent. The disappointing performance was attributed to poor ^1^H channel *B*_1_ homogeneity of the probe. The ratio of the NMR signal intensity at the 90°, 450° and 810° pulse is used as a proxy of an actual *B*_1_ homogeneity measurement. The probes used in this study typically showed I_450_/I_90_ ∼70%, and I_810_/I_90_ ∼55% for the ^1^H channel.

Composite pulses were implemented to compensate for the probe deficiencies since the standard symmetry recoupling experiments were unsuccessful. Two composite-π pulses are prominent in the literature, the [90_(0)_90_(90)_90_(0)_] and [90_(0)_270_(180)_] where the flip angle of a portion of the pulse is denoted by the large number and the phase of that portion is denoted by the subscripted number in parentheses (Levitt, 2002). The comparison of the scaling factors determined *in silico* by simulations with the SIMPSON program (method described below) shows a dramatic change in the scaling factor when the symmetry element is changed. The performance of the [90_(0)_90_(90)_90_(0)_] composite pulse (Figure 1; Supplementary Figure S1, orange squares) follows the same general trend as the standard π pulse (yellow circles), but with worse efficiency and higher applied field. However, the [90_(0)_270_(180)_] composite pulse (Figure 1; Supplementary Figure S1, red triangles) does not follow the same trend. The shape of the curve produced by the [90_(0)_270_(180)_] composite pulse has a maximum in the curve, whereas the other pulses asymptotically rise. The maximum scaling factor is found at a relatively low field, with performance similar to the π pulse variant.

While it is made clear in the literature that the specifics of the *R*-elements contribute to the efficiency of the recoupling by altering the scaling factor (κ), the magnitude of this contribution was underappreciated. These preliminary simulations demonstrate that more symmetries than those found in the literature should be tried. Those with lower match conditions can be a viable option with composite pulse rotation elements. Also, a variety of symmetry elements will be useful to identify the best candidate sequences given the desired experimental conditions. To these ends, we present tools to generate appropriate symmetry lists, tools to test these symmetries, and experimentally test the best candidates.

### Generating Candidate Symmetry Sequences

An $RN_{n}^{\nu }$ or $CN_{n}^{\nu }$ multiple pulse sequence is applied such that “N” spin-space rotations are contained in “n” sample rotations and the phase (ϕ) of each element alternates as dictated by “ν” where ϕ = ±πν/N for R sequences. This averages some terms of the NMR Hamiltonian to zero, but not others. A brief discussion of the selection rules can be found in the symmetry selection rules section of the supporting information, and in depth in Levitt (2002) and references therein. Each element of the symmetry sequence is a specific rotation where an *R-*element is an inversion (π rotation) and a *C*-element has a 2π total rotation. Therefore, the amplitude for the radiofrequency (ν_1_, *B*_1_) match condition is proportional to the spinning rate and symmetry as

$$\;\upsilon_{1}=k_{p}\frac{\omega_{r}}{2\pi }\frac{N}{2n}$$

where *k*_p_ is determined by the specifics of the *R* or *C*-element.

A custom program was written in Python 3 (see Supplementary Material: SymmetrySelector/SymSelect.py) to generate new symmetry sequences that are more relevant to faster spinning rates. This program reproduces the full list of sequences from Levitt (2002) (excepting 3 minor book-keeping errors, see symmetry selection rules section of the Supplementary Information; Supplementary Tables S2–S4). An arbitrarily large number of candidate symmetry sequences for application at 60 kHz spinning frequency is then generated. Symmetries for most terms in the Hamiltonian can be generated, with the possibility to limit the output based on experimental considerations. 203 symmetries fit the selection criteria (Supplementary Table S5); the sequence must allow heteronuclear dipole-dipole couplings, disallow homonuclear dipole-dipole coupling, and the base applied field is between 0.1 and 150 kHz. It should be noted that this program can be used to generate symmetry sequences for most spin ½ Hamiltonians, not just the heteronuclear dipole-dipole coupling.

### Composite Rotation Element Pulses

Complex inversion pulses have been used for *R*-sequences, such as adiabatic inversions (Herbst et al., 2011; Herbst et al., 2015), numerically optimized optimal control pulses (Nielsen et al., 2009), and composite inversion pulses (Levitt, 1982). However, the *R*-element seems to have previously been chosen for some desirable property of the composite pulse and the expected shortcomings of the symmetry sequence, not explicitly for the performance of the sequence.

Since the timing and phase behavior of the multiple-pulse sequence must still fulfill the symmetry requirements the match field grows in proportion to the total arc swept out by the composite pulse. This term is the pulse contribution to the match condition “*k*_p_,” where:

$$k_{p}=\frac{\sum Sweep\;Angle{}^{\circ}}{180{}^{\circ}}$$

For example, a simple π inversion pulse sweeps an arc of 180°, so *k*_p_ = 1. For the composite pulses [90_(0)_180_(90)_90_(0)_] and [90_(0)_270_(180)_] the RF sweeps out 360°, but the result is only an inversion of the polarization. These specific examples result in *k*_p_ = 2. The match field *B*_1_ is, likewise, twice that of the symmetry alone.

It is unclear which composite pulses produce the highest scaling factor for the smallest applied field. The sweep angles for compound pulses have previously been constrained to only use the principal directions, i.e., they only include integer multiples of 90°, but such a constraint is not enforced in this study. For example, when we modify the [90_(0)_270_(180)_] composite pulse we trade the initial 90_(0)_ portion for a θ_(0)_ pulse, and the 270_(180)_ becomes (180 + θ) _(180)_. The portions of the composite pulse are consecutively numbered τ_1,2…*n*_.The field, timing dependencies, and a diagram of this element, named “1a,” and two others are shown in Table 1. The “2a” element is a slight variation on 1a, it has an extra “wiggle” before finishing. The “4a” element is a variation of the 90_(0)_180_(90)_90_(0)_ element where the middle, out of plane, pulse is allowed to vary. It is possible to change *θ* continuously, and smoothly for these compound pulses and the end point (inversion) will not be changed. In total, 22 *R*-elements were constructed where the sweep angle (and thus the applied field) can be varied continuously, but which always produces a traceable inversion pulse. A simple inversion occurs when *θ* is zero for many, but not all the *R*-elements. All 22 of the *R*-elements with their pulse timings, field match dependence, and a visualization is found in Supplementary Table S6.

**TABLE 1:** Selected variable flip angle *R*-elements, with field and timing dependencies. Red arrows represent the first portion of the composite pulse τ_1_, blue is the second, τ_2_, and yellow is the third portion, τ_3_.

*R*-element	Diagram	Name	k_p_ (B_1_ = k_p_ω_r_N/2n)	τ_1,2,3 …_· (2τ_r_n/N)
θ_(0)_ [180 + θ]_(180)_	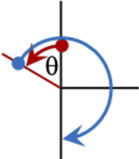	1a	(180 + 2θ)/180	τ_1_ = θ/(180 + 2θ)
				τ_2_= (180 + θ)/(180 + 2θ)
θ_(0)_ [180 + 2θ]_(180)_ θ_(0)_	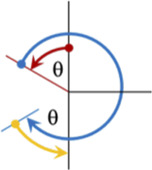	2a	(180 + 4θ)/180	τ_1_ = θ/(180 + 4θ)
				τ_2_= (180 + 2θ)/(180 + 4θ)
				τ_3_ = θ/(180 + 4θ)
90_(0)_ θ_(90)_ 90_(0)_	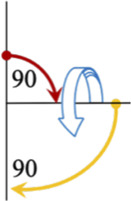	4a	(180 + θ)/180	τ_1_= (90)/(180 + θ)
				τ_2_= (θ)/(180 + θ)
				τ_3_= (90)/(180 + θ)

## Simpson Simulations

Numerical simulations were conducted to determine the scaling factor with the SIMPSON NMR calculation software (Bak et al., 2000). The numerical scaling factor is comparable to the analytical solution presented in Levitt (2002), but differs in magnitude by up to 0.05, where the numerical method always over-estimates the scaling in comparison to the analytical solution. The spin system for testing the scaling factor is an isolated two spin (^1^H-^15^N) system with the dipole-dipole coupling defined to be 10 kHz which corresponds to an internuclear distance of 1.07 Å. The single crystallite crystal file “alpha0beta90” was used so that the maximum dipole-dipole coupling will be produced using the method of Hou et al. (2011) (via personal correspondence).

**FIGURE 2:**
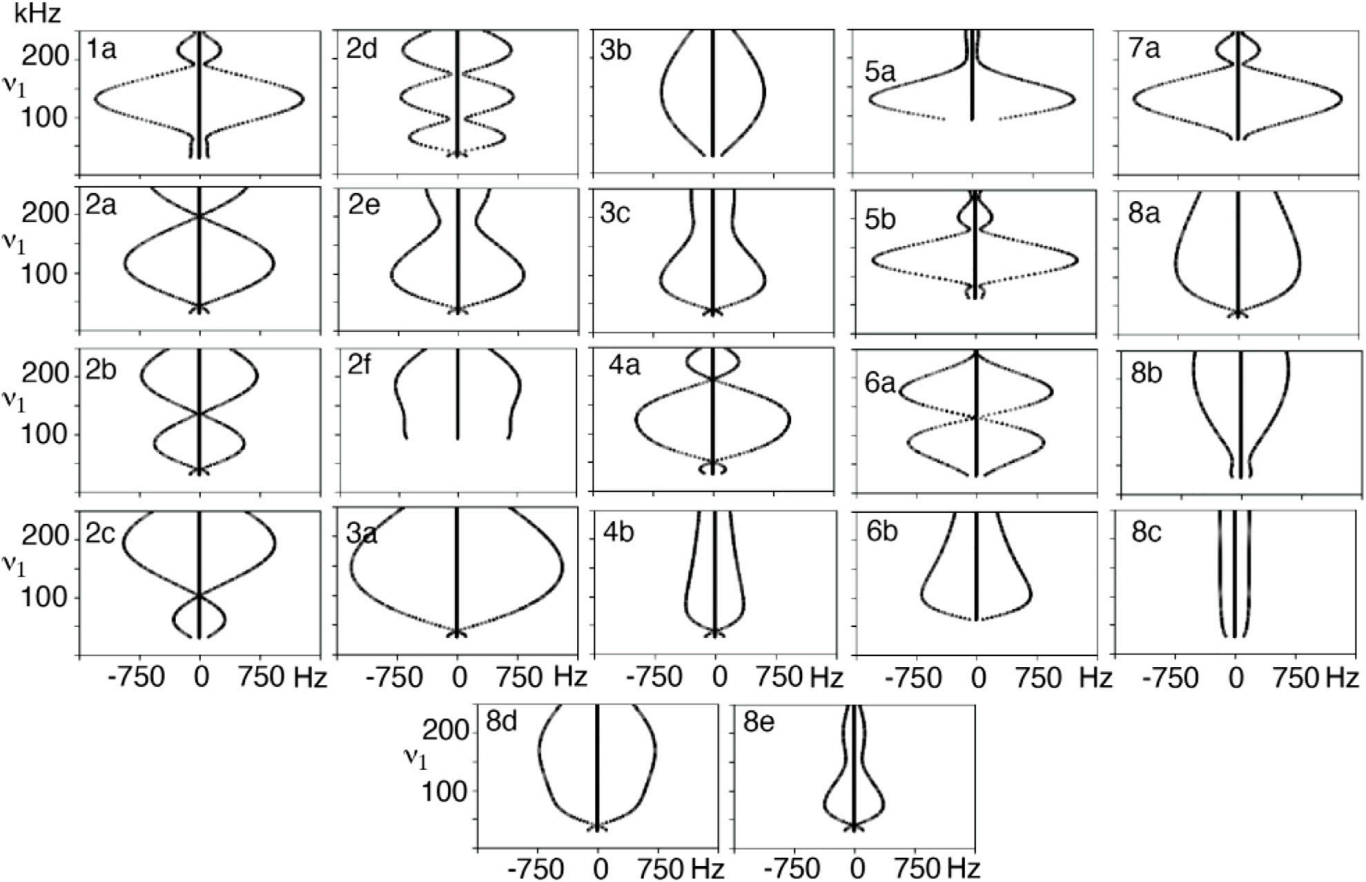
Dipole-dipole recoupling spectra of the R22_21_^9^ symmetry as a function of ν_1_ match condition for different variable flip angle pulse sequence elements.

A series of simulations were performed where the applied field was varied from the lowest match condition up to 250 kHz for the composite *R*-elements to produce dipole recoupling efficiency curves. These curves are well-defined, as demonstrated by the simulations of the symmetry sequence *R*22_21_^9^ in Figure 2. It is convenient to vary the applied field and back calculate the variable flip angles and pulse durations to maintain a valid range for the applied field. The choice of the recoupling element results in decoupling in a sequence which is supposed to recouple, which may point to further selection rules for symmetry elements since there are several instances which result in a coupling of zero. These zero-coupling points might be useful for other applications such as decoupling. These zero points are also present with more crystallites, and thus do not appear to be an artifact of the single crystal simulations. The shape of the curve is an indication of the sensitivity to, for example, *B*_1_ inhomogeneity or a mis-set match condition, where a flatter curve should mean less sensitive condition. There is a significant zero-frequency in the simulated spectra indicating that some fraction of the polarization is not recoupled, although this component is lost when more crystallites are included in the calculation when the calculations are repeated with crystal averaging, as seen in Supplementary Figure S2.

**FIGURE 3:**
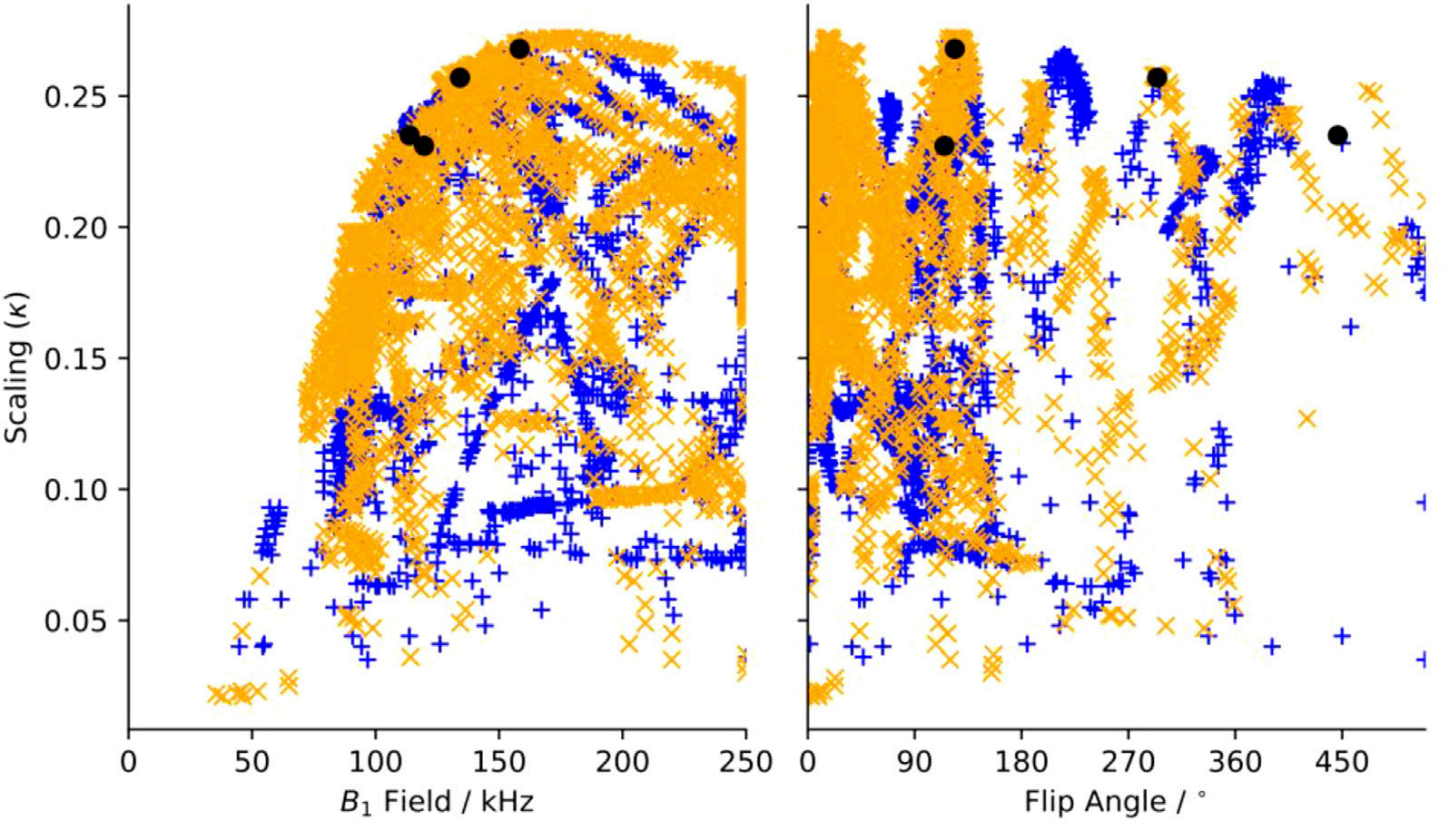
Maximum scaling factor for heteronuclear dipole-dipole scaling factor determined *in silico* of all test symmetries and pulse sequence elements as a function of (a) applied (B_1_) field and of additional tilt angle (θ). Blue“+”’s indicate sequences where “m” and “μ” are correlated, and orange “x”s are anti-correlated. Black circles indicate the position of sequences chosen for experimental verification.

Dipole recoupling efficiency curves are generated for all 22 *R*-elements with 101 applied fields for each of the 203 symmetries. The highest scaling factor for each symmetry and element pair are plotted in Figure 3. Here, the recoupling for the “m” and “μ” (space and spin component) quantum numbers are either correlated (orange X), or anticorrelated (blue +), where this correlation indicates a second order dependency on frequency offset from the carrier (correlated) or on a field mis-set as in *B*_1_ inhomogeneity (anticorrelated), where the frequency offset dependence is the more favorable deficiency.

In Figure 3A, there is a clear limit to the performance as a function of the applied field, where the maximum scaling rises quickly up to a maximum of about κ = 0.27 and ω_1_ = 175 kHz and then slowly reduces as the applied field increases. There are many candidates with κ > 0.225 and ω_1_ < 150 kHz, which are suitable for further testing. When the scaling is plotted against the flip angle, we find that there are certain flip angles that are favored. The first local maximum is at zero, indicating that at least some sequences do not improve using composite pulses. There are local maxima at about 15° and 35°, but the global maximum occurs when there is a flip angle of about 125°, and then approximately every 90° after.

The SIMPSON input files used to evaluate the pulse sequences and Python scripts to process the simulated datasets (i.e., find the maximum in a simple spectrum and report the scaling factor of a 2D simulation) are available online (see *Materials and Methods*).

The 1a element was found to produce the largest scaling factor (or is tied for the largest) for any given symmetry sequence. Additionally, the 1a element has the lowest requirements of any element that performs similarly. That is, the 1a element consistently produces the highest scaling factor for a given symmetry for the least applied field amongst all other competitive R-elements. Therefore, we only considered the 1a element further. The plot of the scaling factors for only the 1a elements as a function applied field and flip angle can be found in Supplementary Figure S3. The intensity of the applied field during the recoupling period is the largest concern, however the maximum duration is short so we felt that we could push the limits of the probe and chose an upper limit of about 150 kHz. Since compensating for poor B_1_ homogeneity was the motivation to use compound pulses, we devised a fast test to show the B_1_, or mis-set, dependence. The 2-spin simulation is run with the optimum flip angle, but the applied field is multiplied by 0.9 or 1.1. About half of the sequences respond strongly and move by hundreds of Hz, while the other half move less than ∼50 Hz. The 0.9 mis-set spectra generally shift more than the 1.1. We found that the mis-set dependence depends on the specific components that are recoupled, specifically if the space component “m” and the spin component “μ” are correlated (both positive or both negative within the same allowed set), the mis-set dependence is typically small. This is due to a “pulse” term in the second order Hamiltonian which is allowed in the anticorrelated set of symmetries and disallowed in the correlated set. The “pulse” term is replaced by a second order frequency offset term in the correlated set of symmetries, which can be demonstrated in the simulations by observing the dependence of the scaling when introducing a frequency offset. This is not an absolute rule, though, since some anti-correlated symmetries are not greatly affected by mis-set, such as R14_8_^5^.

The criteria to choose a symmetry sequence are to find a sequence that works and will not damage either the sample or the hardware. Initially, the criteria were that the maximum applied field should be less than ∼150 kHz, we should use the “1a” element for homonuclear recoupling, the scaling factor should be as large as possible, the spectral width should be large enough that the spectrum does not fold, and the echo time should be short. Ideally, the allowed “m” and “μ” components should be the same sign and the R-element should not stray too far from those previously devised. On close inspection, one notices a small gap at around 150 kHz, after which the scaling factors no longer greatly improve. The sequence immediately after this gap is R20_9_^8^(124_(0)_304_(180)_). Reducing the flip angle to 115° [making R20_9_^8^(115_(0)_295_(180)_)] reduces the applied field to 152 kHz, but does not affect the scaling factor [the scaling is the same down to a 105° flip angle (B_1_ = 145 kHz)]. R20_9_^8^(115_(0)_295_(180)_) thus fulfills all our desired traits. There are many candidate sequences with good scaling, and less demanding match conditions that meet the criteria, but were not tested experimentally. However, the sequences R22_21_^9^ (300_(0)_,480_(180)_) and R14_22_^5^ (460_(0)_,640_(180)_) were chosen to test the robustness of the simulations approach, since the unusual flip-angles in the composite pulses change the scaling factors from almost 0 to above 0.2.

This same *in silico* analysis can, of course, be made under 100 kHz spinning (Supplementary Figure S4). 310 gamma encoded candidate symmetry sequences were generated where *N* = 10 through 42, *n* = 1 through 37, and ν ≤ N/2 and the base match field is limited so that ω_1_ < 200 kHz. The recoupling is less efficient at 100 kHz spinning, as the curve equivalent to the one shown in Figure 3A is shifted to higher match conditions at 100 kHz spinning (Supplementary Figure S4A). The dependence on the flip angle is the same as at 60 kHz spinning, where there are local maxima at ∼15°, 35°, 125°, and then approximately every 90° afterwards (Supplementary Figure S4B). Still, there are several candidate sequences with scaling between κ = 0.15 and κ = 0.20 with relevant match conditions. If we limit the search to the “1a” element, with a match condition of less than ω_1_ = 150 kHz we find 8 candidates, R22_16_^1^(θ = 104), R26_19_^1^(θ = 105), R30_22_^1^(θ = 106), R32_23_^2^(θ = 103), R34_25_^1^(θ = 104), R38_28_^1^(θ = 102), R40_29_^2^(θ = 104), and R42_31_^1^ (θ = 97).

## Experimental Verification

**FIGURE 4:**
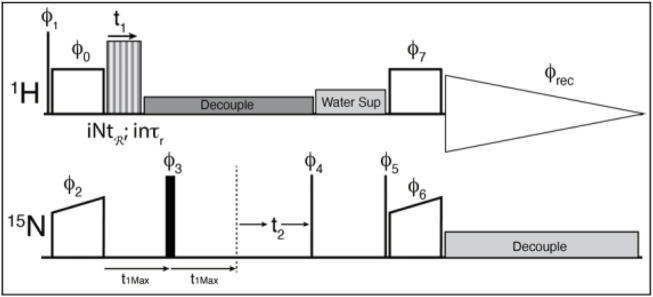
The pulse sequence used for the experiments. Thin lines represent 90° pulses, and thick lines represent 180° pulses, boxes indicate cross polarization spinlock pulses. The sequence is modified from the usual solid-state NMR CP-HSQC by the addition of an echo period, and the application of the recoupling period. ϕ_0_ = 1; ϕ_1_ = 0 0 2 2; ϕ_2_ = 0*4 2*4; ϕ_3_ = 1*8 2*8; ϕ_4_ = 3 1; ϕ_5_ = 1; ϕ_6_ = 0; ϕ_7_ = 1; ϕ_rec_ = 1 3 3 1 3 1 1 3 3 1 1 3 1 3 3 1 where X*4 indicates that phase X is repeated 4 times. The phase shifts for the recoupling experiment are determined by the symmetry sequence and must be adjusted by hand.

A cross-polarization-based ^1^H-detected ^1^H-^15^N correlation experiment was converted into a 3D experiment, where the third dimension consists of a constant time echo period on the low-gamma frequency (Figure 4). The recoupling time increases to create the third, separated local field dimension. The echo period (τ_echo_) is calculated according to the chosen symmetry and data sampling by the following equation:

$$\tau_{echo}=2\times n\tau_{r}\times k\times \left(points-1\right)$$

Where “*n”* is the space winding number from the symmetry sequence, “τ_r_” is the rotor period, “*k*” is the number of repeated symmetry sequences (usually 1) to better fit the spectral width, and “points-1” is the zero-time-point inclusive number of points. We have chosen to apply the recoupling sequence only during the first half of the echo, and a standard decoupling pulse train for the rest of the echo and chemical shift dimensions. That is, the recoupling sequence increments up to the echo pulse, and the echo time is constant throughout the experiment. This is not the ideal case since the echo period should ideally be as short as possible so as little signal is lost as possible. However, the logic needed to loop the symmetry elements before, after and during the π pulse, to properly invert the phases of the symmetry pulses after the echo, and to maintain the proper timings in the Bruker scripting language was too cumbersome, so a simple echo was settled on since the Nitrogen T_2_^*^ is quite long for these samples. It might be possible to use “compound pulse decoupling” pulses to simplify the logic, but we were unsuccessful in our attempts. Alternatively, the π pulse could be used for chemical shift evolution in a constant time evolution experiment, but this will reduce the sensitivity further.

Three recoupling sequences with good theoretical scaling factors and appropriate match conditions were chosen to validate our approach, and to determine which candidate scheme is the most promising. These sequences: R20_9_^8^ (115_(0)_,295_(180)_); R22_21_^9^ (300_(0)_,480_(180)_); and R14_22_^5^ (460_(0)_,640_(180)_) were tested with both their π-pulse version and the numerically optimized sequence elements (Supplementary Figure S5). The sequences were chosen partially because of the diversity of the flip angle, applied field, and the difference in scaling between standard and optimized sequences. The *in-silico* performance of these three sequences, along with R14_8_^5^ (115_(0)_,295_(180)_) are summarized in Table 2. The experimental performance closely follows the *in-silico* performance as demonstrated by strong recoupling with the variable flip angle pulses which lends credence to the approach, and the robustness of symmetry theory in general.

**TABLE 2:** Scaling factor and match conditions for Selected Symmetry sequences.

Symmetry	κ(π)	ν_1_(π) kHz	ν_1_(θ) kHz	κ(θ)	θ in θ_(0)_−(θ + 180) _(180)_
R22_21_^9^	0.0223	31.4	136	0.2567	300°
R14_22_^5^	0.0075	19.1	114	0.2354	460°
R20_9_^8^	0.1354	66.7	152	0.2682	115°
R14_8_^5^	0.0674	52.5	119.5	0.2314	115°

While the R20_9_^8^ (115_(0)_,295_(180)_) seems to be a great option, its match condition is considerable at ν_1_ = 152 kHz. During the power calibration with long nutation experiments, the signal disappeared in a few ms at the fields needed for R20_9_^8^ (115_(0)_,295_(180)_). The probe detuned indicating that such high match conditions would likely damage the sample or the probe, however fields up to about 130 kHz were long lived. The R14_22_^5^ (460_(0)_,640_(180)_) was also tested, but did not produce any recoupling, so the R22_21_^9^ (300_(0)_,480_(180)_) sequence was also thrown out since both have quite long echo times, and quite large flip angles. Other candidate sequences were identified from the scaling curve that have more conventional *R*-elements and lower match conditions. Amongst a handful of candidate sequences, the R14_8_^5^ (115_(0)_,295_(180)_) sequence was the first one that worked (it was also the first one tested). The R14_8_^5^ (115_(0)_,295_(180)_) has a lower match condition than the R20_9_^8^(115_(0)_,295_(180)_), the R-element is not far from a canonical R-element, and it seems to have a high tolerance for field mis-set in numerical simulations. The match condition (ν_1_ = 119.5 kHz) for R14_8_^5^ (115_(0)_,295_(180)_) is very near the ν_1_/2p = 2ω_r_ rotary resonance condition, although this does not seem detrimental to the quality of the data in the microcrystalline sample. If the match is a greater concern, the *R*-element could be adjusted for a lower match condition as it was for the R20_9_^8^ (115_(0)_,295_(180)_). The scaling factor is the same for all “1a” *R*-elements between θ = 100° and θ = 115°, where ν_1_ ranges from 111.8 to 119.5 kHz. The ability to turn down the power was not appreciated at the time the experiment was conducted.

### Temperature Dependent Order Parameters

To validate the designed experiments, we have first applied them to a favorable model sample of crystalline 100% H_2_O [U-^2^H,^13^C,^15^N]GB1, which means that the protein is uniformly ^13^C and ^15^N labelled and perdeuterated with only exchangeable protons being reintroduced at 100%. We used R14_8_^5^ (115_(0)_,295_(180)_) sequence to measure ^15^N-^1^H order parameters at three different temperatures, nominally 263.2, 273.2, and 283.2 K, which correspond to sample temperatures of ∼302 K, ∼309 and ∼315 K (larger temperature differences are difficult to obtain on our 1.3 mm probes at 60 kHz spinning; see *Materials and Methods*). A representative ^1^H-^15^N correlation spectrum taken from the first plane of the 3D can found in the Supplementary Figure S9.

**FIGURE 5:**
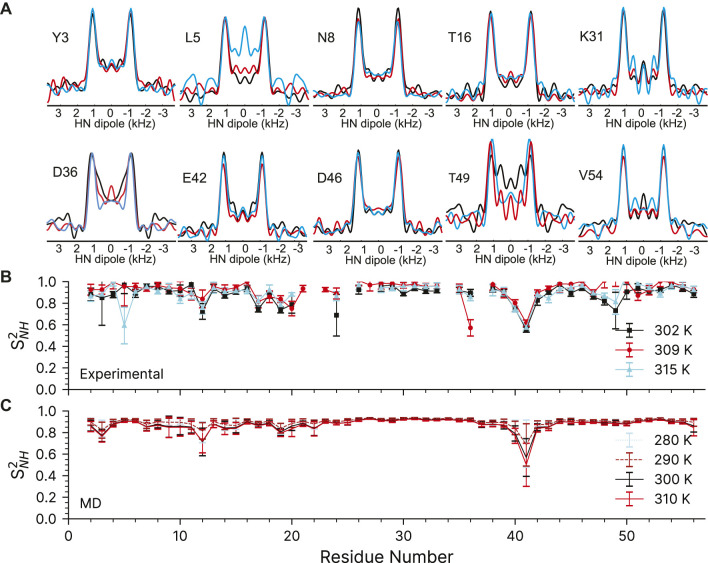
^15^N-^1^H dipolar couplings as a function of temperature in crystalline GB1. **(A)** Representative dipole-dipole recoupling spectra using the R14_8_^5^ (115_(0)_,295_(180)_) pulse sequence at ∼302 K, ∼309 and ∼315 K at 60 kHz spinning. **(B)** S^2^_NH_ obtained from the measured dipolar couplings, error bars are drawn at 1σ. **(C)** S^2^_NH_ obtained from variable temperature molecular dynamics simulations. MD derived order parameters were extracted from 400 ns molecular dynamics simulations of a 3 × 3 × 3 supercell of GB1 containing 108 monomers.

The quality of the recoupled line-shapes for all temperatures is excellent as evidenced in Figure 5A. There is surprisingly little intensity at the zero-frequency, and the dipole line-shape is clear and strong. As expected, there is little difference in the experimentally observed (fitted) coupling over the explored temperature range and the obtained ^15^N-^1^H order parameters are generally very similar for all three measurements (see Supplementary Table S7). We only observe a noticeable change of ^15^N-^1^H dipole-dipole coupling (from 11.1 to 10.2 kHz) as a function of temperature for L5, D36, and T49 which are low intensity peaks in the 2D fingerprint spectrum. In the ∼309 K data, the order parameter for D36 is spuriously low for reasons which are not clear (See Supplementary Figure S8). The precision of the experiment is very good: the 1σ standard deviation in the dipole couplings is around ±220 Hz, with some down to ±100 Hz, and outliers ranging from ±500 to 1500 Hz. These errors are comparable to previous symmetry-based methods on the same protein (Franks et al., 2005; Franks et al., 2006b). However, the precision may be overstated due to poor noise estimation in the Monte Carlo error analysis. Monte Carlo error analysis first finds the best fit for the experimental data to a simulation, here the time-domain dipole-dipole recoupling curve. Noise is then added to each point of the experimental data and the best fit is found again, and the values saved. Noise is added to the original experimental data several times (here, 5,000 times) to estimate the amount of spread in the simulation values found in the experimental data. The method we have used to add the noise could be improved. First, most peaks have very similar initial intensities, so a constant noise value was used for all peaks in the Monte Carlo analysis, which was ±7.5% of the total. All trajectories were normalized to 1 during the integrations, which results in an undesirable loss of information regarding the intensity. The result is that a resonance that is 100 intensity units high will have noise ranging from −7.5 to +7.5 added, while a peak that is only 10 Intensity units high will only have noise added that ranges from −0.75 to +0.75 units. The noise estimate works well for peaks with a typical intensity, and may even be larger than necessary, but the error analysis fails for peaks with poor intensity. A typical Monte Carlo fit is shown for residue K28 in Supplementary Figure S6. Similar figures for all fits can be found in their corresponding datasets in the online materials. Those residues with worse sensitivity will have spuriously good fits, such as found for the K13 peak in the GB1+IgG complex (Supplementary Figure S8).

The ^15^N-^1^H dipolar order parameters shown in Figure 5B fit well with those reported previously (Franks et al., 2005; Franks et al., 2006b). The ^15^N-^1^H dipolar order parameters are generally around 0.9 (order parameters, S^2^, take values between 0 and 1, which mean unrestricted motion and no motion respectively) with an occasional dip to around 0.8 near the loops. The largest amplitudes of motions are observed for residue G41, which is in a loop between the alpha helix and beta strand 3.

We have also compared the experimentally determined ^15^N-^1^H order parameters to those obtained from molecular dynamics simulations performed in the 280–310 K temperature range (Figure 5C). The simulated rates are in general good agreement with the experimental ones (see Supplementary Figure S7). As before (Busi et al., 2018), the MD simulations predict that there should be little change in the order parameters of the expected temperature range.

## Order Parameters of the GB1-IgG Complex

The complex of 100% H_2_O [U-^2^H,^13^C,^15^N]GB1 with IgG is a much more challenging sample compared to crystalline GB1 to apply the described methods. As a precipitate it is more heterogenous, >90% of the sample volume is taken by the antibody resulting in lower sensitivity and GB1 in the complex exhibits much more pronounced slow motions in the microsecond range. (Lamley et al., 2014; Lamley et al., 2015a; Öster et al., 2019). A paramagnetic doping agent, 2 mM Gd (DTPA-BMA), was added to speed up the measurements by reducing the required relaxation delays (Linser et al., 2007), although in this particular application sample heating is a large concern so the recovery delay remains quite long at 1s. The ^1^H-^15^N correlation spectrum taken from the first plane of the 3D can found in the Supplementary Figure S9.

**FIGURE 6:**
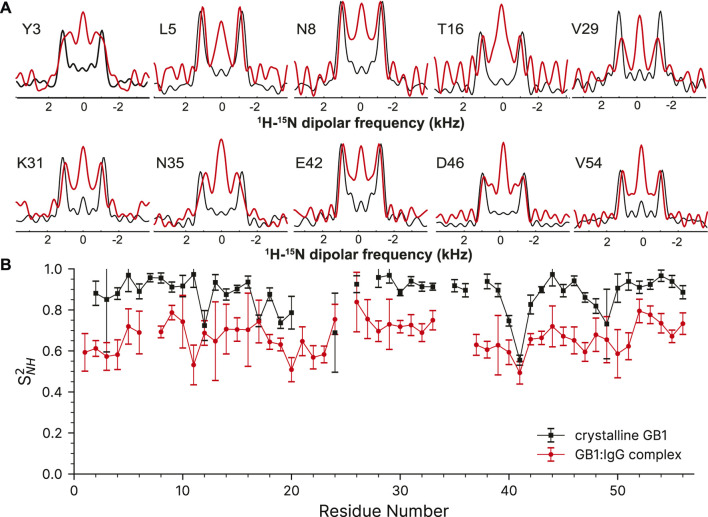
^15^N-^1^H dipolar order parameter data for GB1 in a crystal (black line) and in a precipitated complex with IgG (red line). **(A)** Overlay of representative dipole recoupling spectra. **(B)** ^15^N-^1^H order parameters as a function of residue, error bars are drawn at 1σ. Both data sets were recorded at the nominal temperature of 263.2 K corresponding to sample temperature of ∼302 K at 60 kHz spinning frequency.

Despite the more challenging nature of the sample, the GB1 in the complex still produces high-quality dipole-recoupling spectra, as seen in Figure 6A. There is a significant zero-frequency component in most of these spectra, and the sensitivity is generally worse (especially for T16). The origin of the zero-frequency component is possibly due to the larger amount of ^1^H atoms in the sample, less efficient ^1^H-^1^H homo-decoupling, increased dynamics, sample heating, and/or probe detuning. The determined ^15^N-^1^H order parameters are generally lower in the GB1 complex (average S^2^_NH_ ∼0.7) than in crystalline GB1 (average S^2^_NH_ ∼0.9). If one uses expression for order parameter in diffusion in a cone model this difference corresponds on average to ∼23° additional motional amplitude for most residues. This provides further support for presence of a microsecond range overall motion of GB1 in the complex with IgG as proposed previously (Lamley et al., 2015a).

## Conclusion

We have presented a method to apply symmetry-based recoupling theory to fast MAS experiments using variable flip angle compound pulses. The method generates many candidate sequences that have a reasonably high scaling factor and applicable match conditions. Being able to apply symmetry principles under fast-MAS makes SLF methods applicable to faster spinning and the other benefits that usually comes with it. The heteronuclear dipole-dipole coupling can be measured site-specifically in microcrystalline GB1 and in the GB1-IgG complex. The order parameters found using the variable flip angle method are consistent with previous datasets and molecular dynamics in the microcrystalline sample. The GB1 in the complex shows both localized differences in dynamics and global increase of cumulative motional amplitudes.

## Materials and Methods

### Sample Preparation

Uniformly (^2^H, ^13^C, ^15^N) GB1 was produced as described previously (Franks et al., 2005; Zhou et al., 2007b). After production in ^2^H buffer with ^13^C glucose and ^15^N NH_4_Cl, the protein is placed in ^1^H containing buffer, and heated so that the exchangeable ^1^H sites are ^1^H labelled. The protein is then either crystallized or incubated with natural abundance IgG in an equimolar ratio (Lamley et al., 2014). All back-bone amide sites on the GB1 molecules are thus labeled, but the strong ^1^H dipole coupling network is disrupted locally by the ^2^H labelling of the sidechain carbons. The buffer of the IgG-GB1 complex contained 2 mM of paramagnetic Gd (DTPA-BMA)*.*

### NMR Spectroscopy

All experiments were performed on either a Bruker Avance III spectrometer at 700.13 MHz ^1^H Larmor frequency or a Bruker Avance II spectrometer or at a 599.4 MHz ^1^H Larmor frequency. A Bruker 1.3 mm HCN Probe operating in HCN triple resonance mode with a sample spinning rate of 60 kHz ± 3 Hz was used with both instruments. 1,200 L/h of cooling gas was used at the nominal temperatures of 263.2, 273.2, and 283.2 K. The nutation frequencies for the 90° pulses were calibrated so that the hard pulses for ^1^H were 2.1 μs (*ν*_1_ = 120 kHz); ^13^C, 2.5 μs (*ν*_1_ = 100 kHz); and ^15^N, 3.25 μs (*ν*_1_ = 77 kHz). The ^1^H carrier radiofrequency (RF) was centred on the H_2_O signal (∼4.7 ppm), ^15^N at 120 ppm, and ^13^C at 100 ppm. Heteronuclear ^1^H decoupling (∼10 kHz SPINAL-16) (Fung et al., 2000) was used during the indirect chemical shift dimension, and during the echo period when the recoupling sequence was not being applied, and approximately 10 kHz WALTZ-64 (Zhou et al., 2007c) ^15^N decoupling was used during ^1^H acquisition. The States-TPPI method was employed for quadrature detection in the indirect chemical shift dimension (Marion et al., 1989) and only the real portion of the dipole-coupling dimension is acquired. The MISSISSIPPI (Zhou and Rienstra, 2008) solvent suppression scheme was applied with a spinlock field of ∼30 kHz for four 15 ms intervals before detection. The symmetry match condition was calibrated to the theoretical value by varying the applied power in a 2D, nitrogen edited, ^1^H nutation experiment until the nutation experiment is within 1 Hz.

The total experiment time for each temperature point of the crystalline GB1 was 16 h each ^1^H free induction decay was acquired for 40 ms with a spectral width of 30 ppm with 32 coadded transients. The ^15^N dimension for the microcrystalline protein were acquired with 80 rows with a dwell of 333.33 µs for a total of 13.3 ms in the indirect dimensions. The R14_8_^5^ (115_(0)_,295_(180)_) dimension was acquired for 15 real points with an increment of 8*τ_r_ = 133.33 µs for a total of 1.87 ms (3.73 ms total echo time). The recovery delay was 1.5 s.

The spectrum of the GB1-IgG complex was collected in four blocks of 34.1 h each which were later summed together, for a total of 5 days 16.5 h. There was 1,200 L/h of variable temperature gas flow at the nominal temperature of 263.2 K. Each ^1^H free induction decay was acquired for 30 ms with a spectral width of 30 ppm in four blocks of 128 coadded transients (512 total), the ^15^N dimension for the microcrystalline protein were acquired with 64 rows with a dwell of 333.33 µs, with a spectral width of 42 ppm (^15^N) for a total of 10.7 ms in the indirect dimensions, the R14_8_^5^ (115_(0)_,295_(180)_) dimension was acquired for 15 real points with an increment of 8*τ_r_ = 133.33 µs for a total of 1.87 ms (3.73 ms total echo time) with a relaxation delay of 1 s All 3D data was processed using NMRPipe and the four blocks were added using NMRPipe (Delaglio et al., 1995). The dipole-recoupling dimension was not Fourier-transformed in NMRPipe so that peak volumes could be extracted. The Fourier-transform of the dipole recoupling dimension was performed on the peak volumes extracted by NMRPipe by a the fast fourier transform routine found in python’s numpy package. The imaginary components of the trajectories were filled with zeroes, and the trajectory was zero filled to 512 points. The 2D datasets with a dipole recoupling dimension (Supplementary Figure S5) were processed in Topspin with by performing a Hilbert-transform to fill the imaginary portion of the dipole trajectory before Fourier transforming the dipole recoupled dimension. (See Supplementary Material: BrukerMacros/2DHN).

External KBr (Thurber and Tycko, 2009) and neat methanol (Ammann et al., 1982) were used as external standards to calibrate the temperature. The samples did not have adequate resolution to unambiguously identify the bulk water signal from the isopropanol and methyl-pentane-diol OH signals, which precluded temperature calibration by the chemical shift difference between water and DSS (Hoogen et al., 1988; Wishart et al., 1995). Temperatures derived using either the chemical shift or the T_1_ of KBr (Thurber and Tycko, 2009) were not self-consistent on the 700 MHz instrument at either 10 or 60 kHz spinning. There is approximately 10°C difference between the two methods. The T_1_ method indicates a 20°C difference across the nominal temperatures, where the chemical shift method indicates a difference of 13.6°C. Calibrating the temperature by the chemical shift difference in the ^1^H spectrum of methanol (Ammann et al., 1982) indicates temperatures of 301.9 (263.2), 309.0 (273.2), and 314.9 K (283.2) or 28.7, 35.8, and 41.7°C under the experimental conditions, for a range of 12.9°C.

### Data Processing, Fitting and Simulations

The GB1 resonances were identified in the 2D finger-print spectra from the literature and previous work (Franks et al., 2005; Zhou et al., 2007a). Peaks were integrated using NMRPipe (Delaglio et al., 1995), where the peak volumes were converted into dipole recoupling trajectories. The volume of the peak on the first plane is used to normalize the rest of the curve, so all curves range between ±1, and start with an intensity of 1.00. Overlapping resonances were fit, but these resonances were not included in the figures or analysis since the resonance could not be unambiguously identified.

A library of numerical simulations was created in SIMPSON 4.2.1 on an Apple MacBook Pro for use in the Monte-Carlo fitting routine. The simulation library was created using 251 dipole-dipole couplings ranging from 7,500 Hz to 12,500 Hz in steps of 20 Hz, the spin rate was 60 kHz, the calculation method was “direct”, the crystal file was “zcw376,” and 16 gamma angles were used. The time-domain trajectories and frequency domain spectra were saved as a 2D SIMPSON file.

5000 Monte Carlo steps were used for error analysis for all datasets only using the time domain SIMPSON library. The library was expanded using simple operations on the time-domain. The DC parameter is used to add a constant is to all data points (varied between −0.2 and +0.5). The scaling factor multiplies each point by a constant (varied from 0.9 to 1.1). Finally, relaxation is simulated by applying line-broadening for each trajectory, that is, the simulation is multiplied by a time dependent exponential function (from 0 Hz to 2,500 Hz). To Fourier transform the dipole trajectories, the imaginary time portion was filled with zeroes, and the trajectory was zero-filled to 128 points. For the crystalline GB1, most line shapes fit with a small, negative DC offset (−0.05), a scaling multiplier of 1.00, and less than 300 Hz of line-broadening. For the IgG-GB1 complex, there is a small positive DC offset (+0.14), a scale of 1.00, and approximately 600 Hz of line-broadening on average. The rigid limit for the N-H dipolar coupling is taken as 11,477.3 Hz to determine the Order Parameters, which corresponds to an N-H bond length of 1.02 Å.

### Molecular Dynamics Simulations

A molecular dynamics trajectory for a 3 × 3 × 3 supercell of GB1 containing 108 monomers was computed using AMBER MD (Case et al., 2005; Doshi and Hamelberg, 2009; Maier et al., 2015; Tian et al., 2019). The coordinates of the X-ray structure of GB1 (PDB:2gi9 Franks et al., 2006b) were taken as a starting conformation. To the supercell, 108 PO_4_^3−^ counter ions were added. 12,852 explicit water molecules were added, followed by charge balancing with sodium ions giving an overall box size of 75.591 Å × 107.152 Å × 150.822 Å. The ff19SB (Tian et al., 2019) forcefield was used for the GB1 proteins, with OPC water (Izadi et al., 2014) and GAFF cocrystallites (Wang et al., 2004). After minimization, the system was replicated and heated to the temperatures indicated in the figures (280, 290, 300, 310 K). The systems were then simulated for full 400 ns runs. For each, a 2 fs timestep was used with a cut-off of 11 Å for non-bonded interactions. Temperatures were maintained using a Langevin thermostat, and the SHAKE algorithm (Ryckaert et al., 1977) was applied to all bond lengths involving a hydrogen atom. Anisotropic pressure scaling was used with periodic boundary conditions.

Prior to processing, the Cα carbons between timesteps were aligned using cpptraj (Roe and Cheatham, 2013). Then, correlation functions for each N-H vector were calculated according to the iRED framework using cpptraj (Prompers and Brüschweiler, 2002). The median was calculated for each residue over all GB1s in the supercell for which order parameters could be extracted, and the error taken as twice the median absolute difference.

## Data Availability

The datasets presented in this study can be found in online repositories. The names of the repository/repositories and accession number(s) can be found in the article/Supplementary Material.
