# Supplementary material for: Dipolar Order Parameters in Large Systems With Fast Spinning
Source: Front Mol Biosci. 2021 Dec 9;8:791026. doi: 10.3389/fmolb.2021.791026 (PMC8699854; doi:10.3389/fmolb.2021.791026)
Supplement: Supplementary file 1 [file Table10.docx]

| **Residue** | **Dipolar coupling (Hz)**  **T_nom_ = 263 K, GB1** | | **Dipolar coupling (Hz)**  **T_nom_ = 273 K, GB1** | | **Dipolar coupling (Hz)**  **T_nom_ = 283 K, GB1** | | **Dipolar coupling**  **(Hz)**  **T_nom_ = 263 K, GB1:IgG** | |
| --- | --- | --- | --- | --- | --- | --- | --- | --- |
| Q2 | 10775 | ±362 | 11041 | ±298 | 10680 | ±301 | 8839 | ±684 |
| Y3 | 10588 | ±1591 | 11047 | ±253 | 10782 | ±161 | 8980 | ±282 |
| K4 | 10764 | ±173 | 11447 | ±358 | 11494 | ±247 | 8689 | ±512 |
| L5 | 11299 | ±467 | 11217 | ±1058 | 8839 | ±1265 | 8755 | ±544 |
| I6 | 10831 | ±221 | 11210 | ±178 | 10948 | ±189 | 9735 | ±582 |
| L7 | 11229 | ±117 | 11063 | ±106 | 11193 | ±113 | 9533 | ±720 |
| N8 | 11220 | ±144 | 11276 | ±160 | 10972 | ±166 |  |  |
| G9 | 10958 | ±149 | 11145 | ±221 | 11189 | ±195 | 9553 | ±197 |
| K10 | 10988 | ±312 | 11112 | ±382 | 10517 | ±257 | 10182 | ±227 |
| T11 | 11322 | ±367 | 10798 | ±338 | 10853 | ±343 | 9891 | ±855 |
| L12 | 9764 | ±492 | 10518 | ±519 | 9993 | ±372 | 8370 | ±762 |
| K13 | 11094 | ±225 | 11225 | ±193 | 11151 | ±302 | 9516 | ±415 |
| G14 | 10736 | ±167 | 11040 | ±267 | 10930 | ±216 | 9237 | ±1369 |
| E15 | 10901 | ±110 | 11150 | ±144 | 10972 | ±132 | 9647 | ±828 |
| T16 | 11103 | ±174 | 11473 | ±228 | 11018 | ±199 | 9633 | ±390 |
| T17 | 9918 | ±188 | 10355 | ±360 | 10382 | ±307 | 9625 | ±1220 |
| T18 | 10745 | ±191 | 10642 | ±335 | 11044 | ±254 | 9896 | ±694 |
| E19 | 9854 | ±181 | 10506 | ±198 | 10169 | ±208 | 9210 | ±259 |
| A20 | 10179 | ±515 | 9943 | ±447 | 10776 | ±96 | 9119 | ±223 |
| V21 |  |  | 11099 | ±176 |  |  | 8187 | ±473 |
| D22 |  |  |  |  |  |  | 9233 | ±501 |
| A23 |  |  | 11044 | ±116 |  |  | 8656 | ±432 |
| A24 | 10144 | ±181 | 10832 | ±307 | 10680 | ±255 | 8763 | ±306 |
| T25 |  |  |  |  |  |  | 9967 | ±485 |
| A26 | 11041 | ±245 | 11533 | ±283 | 11319 | ±279 |  |  |
| E27 |  |  | 11296 | ±322 |  |  | 10509 | ±910 |
| K28 | 11236 | ±284 | 11387 | ±313 | 11164 | ±275 | 9975 | ±626 |
| V29 | 11298 | ±222 | 11386 | ±239 | 11154 | ±168 | 9577 | ±348 |
| F30 | 10795 | ±92 | 11274 | ±157 | 11243 | ±261 | 9805 | ±823 |
| K31 | 11128 | ±132 | 11351 | ±85 | 11250 | ±148 | 9728 | ±205 |
| Q32 | 10969 | ±133 | 11210 | ±174 | 11199 | ±166 | 9781 | ±344 |
| Y33 | 10967 | ±101 | 11250 | ±156 | 11241 | ±176 | 9528 | ±299 |
| A34 |  |  |  |  |  |  | 9939 | ±312 |
| N35 | 10997 | ±179 | 11117 | ±234 | 11055 | ±230 |  |  |
| D36 | 10864 | ±196 | 8676 | ±575 | 10635 | ±219 |  |  |
| N37 |  |  |  |  | 11025 | ±321 |  |  |
| G38 | 11121 | ±211 | 11327 | ±312 | 10937 | ±233 | 9108 | ±368 |
| V39 | 10866 | ±194 | 10852 | ±265 | 9905 | ±176 | 8937 | ±299 |
| D40 | 9918 | ±170 | 10279 | ±197 | 8697 | ±198 | 9096 | ±1048 |
| G41 | 8550 | ±187 | 9004 | ±213 | 10775 | ±133 | 8843 | ±445 |
| E42 | 10435 | ±290 | 10870 | ±161 | 11093 | ±572 | 8064 | ±448 |
| W43e | 10202 | ±853 | 11076 | ±518 | 10566 | ±267 | 9304 | ±192 |
| W43 | 10882 | ±87 | 11130 | ±108 | 11096 | ±257 |  |  |
| T44 | 11322 | ±327 | 11357 | ±485 | 11165 | ±161 | 9351 | ±228 |
| Y45 | 10851 | ±182 | 11349 | ±217 | 11268 | ±178 | 9737 | ±675 |
| D46 | 11141 | ±139 | 11496 | ±223 | 10852 | ±117 | 9405 | ±313 |
| D47 | 10648 | ±142 | 10859 | ±180 | 10702 | ±147 | 9265 | ±467 |
| A48 | 10386 | ±225 | 11535 | ±389 | 10341 | ±731 | 8853 | ±340 |
| T49 | 9816 | ±1137 | 11845 | ±605 | 11943 | ±552 | 9457 | ±568 |
| K50 | 10925 | ±415 | 11485 | ±565 | 11379 | ±318 | 9289 | ±796 |
| T51 | 11121 | ±251 | 10716 | ±283 | 11142 | ±216 | 8790 | ±879 |
| F52 | 10951 | ±181 | 11009 | ±257 | 10832 | ±207 | 9058 | ±476 |
| T53 | 11034 | ±142 | 11498 | ±284 | 11265 | ±201 | 10233 | ±368 |
| V54 | 11284 | ±162 | 11446 | ±262 | 11273 | ±197 | 10112 | ±282 |
| T55 | 11122 | ±172 | 11237 | ±213 | 11035 | ±201 | 9840 | ±316 |
| E56 | 10804 | ±196 | 11051 | ±175 | 10954 | ±107 | 9404 | ±214 |
| The following resonances were not resolved, but could be fit | | | | | | | | |
| V21+E27 | 11015 | ±82 |  |  | 10921 | ±118 |  |  |
| A23+A34 | 11122 | ±97 |  |  | 10680 | ±301 |  |  |
| N37+D22 | 10822 | ±104 | 10975 | ±130 | 10782 | ±161 |  |  |
| D36+A34 |  |  |  |  |  |  | 9942 | ±213 |
