# Supplementary material for: Dipolar Order Parameters in Large Systems With Fast Spinning
Source: Front Mol Biosci. 2021 Dec 9;8:791026. doi: 10.3389/fmolb.2021.791026 (PMC8699854; doi:10.3389/fmolb.2021.791026)
Supplement: Supplementary file 2 [file DataSheet1.zip › TableS8.docx]

Table S7. The dipolar coupling extracted by residue for each experimental condition. The effective sample temperatures for the nominal temperatures of 263 K, 273 K and 283 K were ~302 K, ~309 K and ~315 K, respectively). Error bars reflect errors determined using Monte-Carlo error analysis.

| **Residue** | **Dipolar coupling (Hz)**  **T_nom_ = 263 K, GB1** | | **Dipolar coupling (Hz)**  **T_nom_ = 273 K, GB1** | | **Dipolar coupling (Hz)**  **T_nom_ = 283 K, GB1** | | **Dipolar coupling**  **(Hz)**  **T_nom_ = 263 K, GB1:IgG** | |
| --- | --- | --- | --- | --- | --- | --- | --- | --- |
| Q2 | 10785 | ±120 | 10903 | ±93 | 10675 | ±91 | 9343 | ±258 |
| Y3 | 11215 | ±167 | 10865 | ±131 | 10789 | ±89 | 9361 | ±408 |
| K4 | 10789 | ±112 | 11342 | ±121 | 11396 | ±121 | 9529 | ±220 |
| L5 | 11072 | ±96 | 11081 | ±77 | 10229 | ±213 | 9886 | ±112 |
| I6 | 10860 | ±88 | 11181 | ±95 | 10958 | ±78 | 9772 | ±165 |
| L7 | 11235 | ±91 | 11044 | ±92 | 11185 | ±79 |  |  |
| N8 | 11202 | ±90 | 11327 | ±97 | 10993 | ±102 | 9915 | ±136 |
| G9 | 10946 | ±78 | 11116 | ±87 | 11170 | ±80 | 10234 | ±150 |
| K10 | 11062 | ±86 | 10993 | ±91 | 10579 | ±91 | 10166 | ±184 |
| T11 | 11030 | ±101 | 10824 | ±76 | 10850 | ±89 | 10198 | ±189 |
| L12 | 10106 | ±87 | 10534 | ±103 | 9946 | ±92 | 9531 | ±177 |
| K13 | 11084 | ±82 | 11148 | ±61 | 11054 | ±78 | 11554 | ±173 |
| G14 | 10760 | ±84 | 11050 | ±105 | 10881 | ±89 | 10047 | ±196 |
| E15 | 10945 | ±81 | 11106 | ±82 | 11001 | ±94 | 9782 | ±169 |
| T16 | 11201 | ±71 | 11470 | ±81 | 11022 | ±96 | 9787 | ±300 |
| T17 | 9895 | ±89 | 10490 | ±103 | 10397 | ±114 | 9796 | ±129 |
| T18 | 10821 | ±77 | 10684 | ±93 | 10930 | ±98 | 9469 | ±121 |
| E19 | 9946 | ±86 | 10492 | ±84 | 10093 | ±102 | 9296 | ±138 |
| A20 | 10198 | ±203 | 10123 | ±139 | 10746 | ±98 | 8893 | ±280 |
| V21 |  |  | 11114 | ±95 |  |  | 9333 | ±222 |
| D22 |  |  |  |  |  |  | 9298 | ±154 |
| A23 |  |  | 11096 | ±109 |  |  | 8791 | ±234 |
| A24 | 10526 | ±74 | 10910 | ±142 | 10715 | ±105 | 9810 | ±219 |
| T25 |  |  |  |  |  |  |  |  |
| A26 | 11117 | ±71 | 11383 | ±77 | 11172 | ±77 | 10587 | ±212 |
| E27 |  |  | 11179 | ±79 |  |  | 10128 | ±257 |
| K28 | 11238 | ±96 | 11260 | ±68 | 11204 | ±74 | 9548 | ±108 |
| V29 | 11142 | ±82 | 11267 | ±81 | 11151 | ±86 | 9800 | ±189 |
| F30 | 10780 | ±93 | 11262 | ±97 | 11238 | ±102 | 9720 | ±159 |
| K31 | 11046 | ±84 | 11305 | ±64 | 11165 | ±82 | 9490 | ±212 |
| Q32 | 10943 | ±73 | 11174 | ±86 | 11139 | ±86 | 9704 | ±152 |
| Y33 | 10959 | ±88 | 11282 | ±82 | 11150 | ±88 | 10387 | ±160 |
| A34 |  |  |  |  |  |  | 9094 | ±246 |
| N35 | 11010 | ±79 | 11281 | ±81 | 11096 | ±86 |  |  |
| D36 | 11013 | ±101 | 9112 | ±156 | 10660 | ±109 |  |  |
| N37 |  |  |  |  |  |  |  |  |
| G38 | 11049 | ±50 | 11235 | ±66 | 11042 | ±83 | 9405 | ±167 |
| V39 | 10896 | ±62 | 10838 | ±73 | 10954 | ±83 | 9890 | ±276 |
| D40 | 9886 | ±80 | 10279 | ±111 | 9774 | ±86 | 9201 | ±119 |
| G41 | 8593 | ±53 | 9054 | ±64 | 8742 | ±82 | 8568 | ±75 |
| E42 | 10574 | ±78 | 10920 | ±101 | 10742 | ±102 | 9465 | ±113 |
| W43e | 10357 | ±112 | 11014 | ±81 | 11187 | ±98 |  |  |
| W43 | 10915 | ±78 | 11200 | ±75 | 10561 | ±90 | 9330 | ±230 |
| T44 | 11205 | ±79 | 11293 | ±112 | 11172 | ±76 | 9937 | ±184 |
| Y45 | 10918 | ±102 | 11336 | ±104 | 11178 | ±96 | 9585 | ±180 |
| D46 | 11156 | ±74 | 11420 | ±104 | 11251 | ±101 | 9894 | ±180 |
| D47 | 10648 | ±97 | 10851 | ±107 | 10844 | ±101 | 9078 | ±196 |
| A48 | 10462 | ±89 | 11345 | ±89 | 10714 | ±102 | 9483 | ±510 |
| T49 | 10397 | ±119 | 11511 | ±70 | 10426 | ±110 | 9843 | ±158 |
| K50 | 10813 | ±99 | 11267 | ±140 | 11725 | ±148 | 9722 | ±202 |
| T51 | 11115 | ±67 | 10766 | ±95 | 11260 | ±112 | 9311 | ±196 |
| F52 | 10916 | ±100 | 10973 | ±123 | 11205 | ±94 | 10220 | ±181 |
| T53 | 11056 | ±92 | 11513 | ±120 | 10858 | ±66 | 10109 | ±132 |
| V54 | 11210 | ±84 | 11384 | ±106 | 11202 | ±77 | 9690 | ±180 |
| T55 | 11119 | ±92 | 11247 | ±98 | 11304 | ±99 | 9479 | ±186 |
| E56 | 10742 | ±94 | 11005 | ±75 | 10978 | ±102 | 9810 | ±142 |
| The following resonances were not resolved, but could be fit | | | | | | | | |
| V21+E27 | 11014 | ±77 |  |  |  |  |  |  |
| A23+A34 | 11073 | ±80 |  |  | 10951 | ±92 |  |  |
| N37+D22 | 10814 | ±96 | 10947 | ±91 | 10890 | ±90 |  |  |
| D36+A34 |  |  |  |  |  |  | 9878 | ±205 |
