# Supplementary material for: Dipolar Order Parameters in Large Systems With Fast Spinning
Source: Front Mol Biosci. 2021 Dec 9;8:791026. doi: 10.3389/fmolb.2021.791026 (PMC8699854; doi:10.3389/fmolb.2021.791026)
Supplement: Supplementary file 2 [file DataSheet1.zip › TableS1.docx]

**Table S1.** Symmetry Components

| **Interaction** | **Space rank,**  **l** | **Space component,**  **m** | **Spin Rank,**  **λ** | **Spin Component,**  **μ** |
| --- | --- | --- | --- | --- |
| Isotropic chemical shift | 0 | 0 | 1 | {-1,0,1} |
| Chemical shift anisotropy/Heteronuclear dipole-dipole | 2 | {-2, -1,1,2} | 1 | {-1,0,1} |
| J-coupling | 0 | 0 | 0 | 0 |
| Homonuclear dipole-dipole | 2 | {-2, -1,1,2} | 2 | {-2, -1,0,1,2} |
