# Supplementary material for: Dipolar Order Parameters in Large Systems With Fast Spinning
Source: Front Mol Biosci. 2021 Dec 9;8:791026. doi: 10.3389/fmolb.2021.791026 (PMC8699854; doi:10.3389/fmolb.2021.791026)
Supplement: Supplementary file 2 [file DataSheet1.zip › TableS2.docx]

Table S2. A set of updated *RN_n_^ν^* symmetries as reported in Table 10 of Levitt 2002^34^. The symmetries allow the γ-encoded recoupling of the {l,m,λ,μ} = {2,±2,2,±1} or {2, ±2, 2, ∓1} components of the homonuclear DD couplings with suppression of all other homonuclear DD terms, CSA terms, and isotropic shift terms. The homonuclear J-coupling {0, 0, 0, 0} is symmetry allowed. All inequivalent solutions in the range N ≤ 20, n ≤ 10, and 1 ≤ ν ≤ N/2 are shown. Those symmetries previously reported with ν > N/2 are shown in ***bold italics*** with ν ≤ N/2.

| R10_1_^2^ | R12_1_^2^ | R14_1_^2^ | R16_1_^2^ | R18_1_^2^ | R20_1_^2^ | R14_2_^4^ | R18_2_^4^ | R10_3_^4^ | R14_3_^6^ |
| --- | --- | --- | --- | --- | --- | --- | --- | --- | --- |
| R16_3_^6^ | R20_3_^6^ | ***R14_4_^6^*** | R18_4_^8^ | ***R12_5_^2^*** | ***R14_5_^4^*** | ***R16_5_^6^*** | ***R18_5_^8^*** | ***R14_6_^2^*** | ***R10_7_^4^*** |
| R12_7_^2^ | ***R16_7_^2^*** | ***R18_7_^4^*** | ***R20_7_^6^*** | R14_8_^2^ | ***R18_8_^2^*** | R10_9_^2^ | R14_9_^4^ | R16_9_^2^ | ***R20_9_^2^*** |
| R14_10_^6^ | R18_10_^2^ |  |  |  |  |  |  |  |  |
