# Supplementary material for: Dipolar Order Parameters in Large Systems With Fast Spinning
Source: Front Mol Biosci. 2021 Dec 9;8:791026. doi: 10.3389/fmolb.2021.791026 (PMC8699854; doi:10.3389/fmolb.2021.791026)
Supplement: Supplementary file 2 [file DataSheet1.zip › TableS3.docx]

Table S3. A set of updated *RN_n_^ν^* symmetries as reported in Table 12 of Levitt 2002^34^. The symmetries allow the selection of homonuclear J-couplings {l,m,λ,μ} = {0,0,0,0} with suppression of all other homonuclear DD terms, CSA terms, and isotropic shift terms. All inequivalent solutions in the range 2 ≤ N ≤ 20, 1 ≤ n ≤ 10, and 0 ≤ ν ≤ N/2 are shown. Those symmetries that not found in the literature^34^ with n >5, are shown in ***bold italics****.*

| R6_1_^0^ | R8_1_^0^ | R10_1_^0^ | R12_1_^0^ | R12_1_^3^ | R14_1_^0^ | R14_1_^4^ | R16_1_^0^ | R16_1_^3^ | R16_1_^3^ |
| --- | --- | --- | --- | --- | --- | --- | --- | --- | --- |
| R16_1_^4^ | R16_1_^5^ | R18_1_^0^ | R18_1_^3^ | R18_1_^4^ | R18_1_^5^ | R18_1_^6^ | R20_1_^0^ | R20_1_^3^ | R20_1_^4^ |
| R20_1_^5^ | R20_1_^6^ | R20_1_^7^ | R6_2_^0^ | R10_2_^0^ | R12_2_^3^ | R14_2_^0^ | R16_2_^3^ | R16_2_^5^ | R18_2_^0^ |
| R18_2_^3^ | R18_2_^6^ | R20_2_^3^ | R20_2_^5^ | R20_2_^7^ | R8_3_^0^ | R10_3_^0^ | R14_3_^0^ | R14_3_^2^ | R14_3_^5^ |
| R16_3_^0^ | R16_3_^1^ | R16_3_^4^ | R16_3_^7^ | R18_3_^1^ | R18_3_^2^ | R18_3_^4^ | R18_3_^5^ | R18_3_^7^ | R18_3_^8^ |
| R20_3_^0^ | R20_3_^1^ | R20_3_^2^ | R20_3_^5^ | R20_3_^7^ | R20_3_^8^ | R20_3_^9^ | R6_4_^0^ | R10_4_^0^ | R12_4_^1^ |
| R12_4_^3^ | R12_4_^5^ | R14_4_^0^ | R18_4_^0^ | R18_4_^3^ | R18_4_^6^ | R20_4_^1^ | R20_4_^3^ | R20_4_^5^ | R20_4_^7^ |
| R20_4_^9^ | R6_5_^0^ | R8_5_^0^ | R12_5_^0^ | R12_5_^3^ | R14_5_^0^ | R14_5_^1^ | R14_5_^6^ | R16_5_^0^ | R16_5_^4^ |
| R16_5_^7^ | R18_5_^0^ | R18_5_^2^ | R18_5_^3^ | R18_5_^6^ | R18_5_^7^ | ***R10_6_^0^*** | ***R14_6_^0^*** | ***R16_6_^1^*** | ***R16_6_^7^*** |
| ***R18_6_^1^*** | ***R18_6_^2^*** | ***R18_6_^4^*** | ***R18_6_^5^*** | ***R18_6_^7^*** | ***R18_6_^8^*** | ***R20_6_^1^*** | ***R20_6_^5^*** | ***R20_6_^9^*** | ***R6_7_^0^*** |
| ***R8_7_^0^*** | ***R10_7_^0^*** | ***R12_7_^0^*** | ***R12_7_^3^*** | ***R16_7_^0^*** | ***R16_7_^3^*** | ***R16_7_^4^*** | ***R16_7_^5^*** | ***R18_7_^0^*** | ***R18_7_^1^*** |
| ***R18_7_^3^*** | ***R18_7_^6^*** | ***R18_7_^8^*** | ***R20_7_^0^*** | ***R20_7_^1^*** | ***R20_7_^2^*** | ***R20_7_^5^*** | ***R20_7_^8^*** | ***R20_7_^9^*** | ***R6_8_^0^*** |
| ***R10_8_^0^*** | ***R12_8_^1^*** | ***R12_8_^3^*** | ***R12_8_^5^*** | ***R14_8_^0^*** | ***R18_8_^0^*** | ***R18_8_^3^*** | ***R18_8_^6^*** | ***R20_8_^1^*** | ***R20_8_^3^*** |
| ***R20_8_^5^*** | ***R20_8_^7^*** | ***R20_8_^9^*** | ***R8_9_^0^*** | ***R10_9_^0^*** | ***R14_9_^0^*** | ***R14_9_^1^*** | ***R14_9_^6^*** | ***R16_9_^0^*** | ***R16_9_^3^*** |
| ***R16_9_^4^*** | ***R16_9_^5^*** | ***R20_9_^0^*** | ***R20_9_^3^*** | ***R20_9_^4^*** | ***R20_9_^5^*** | ***R20_9_^6^*** | ***R20_9_^7^*** | ***R6_10_^0^*** | ***R12_10_^3^*** |
| ***R14_10_^0^*** | ***R16_10_^1^*** | ***R16_10_^7^*** | ***R18_10_^0^*** | ***R18_10_^3^*** | ***R18_10_^6^*** |  |  |  |  |
