# Supplementary material for: Dipolar Order Parameters in Large Systems With Fast Spinning
Source: Front Mol Biosci. 2021 Dec 9;8:791026. doi: 10.3389/fmolb.2021.791026 (PMC8699854; doi:10.3389/fmolb.2021.791026)
Supplement: Supplementary file 2 [file DataSheet1.zip › TableS4.docx]

Table S4. A set of updated *RN_n_^ν^* symmetries as reported in Table 14 of Levitt 2002^34^. The symmetries allow the isotropic chemical shift terms {l,m,λ,μ} = {0,0,1,±1} with suppression of all other homonuclear DD terms, and CSA terms. The homonuclear J-coupling {0, 0, 0, 0} is symmetry allowed. All inequivalent solutions in the range 2 ≤ N ≤ 20, 1 ≤ n ≤ 10, and 0 ≤ ν ≤ N/2 are shown. Those symmetries not found in the literature^34^ with n >5, are shown in ***bold italics****.*

| R6_1_^3^ | R8_1_^4^ | R10_1_^5^ | R12_1_^6^ | R14_1_^7^ | R16_1_^8^ | R18_1_^9^ | R20_1_^10^ | R6_2_^3^ | R10_2_^5^ |
| --- | --- | --- | --- | --- | --- | --- | --- | --- | --- |
| R14_2_^7^ | R18_2_^9^ | R8_3_^4^ | R10_3_^5^ | R14_3_^7^ | R16_3_^8^ | R20_3_^10^ | R6_4_^3^ | R10_4_^5^ | R14_4_^7^ |
| R18_4_^9^ | R6_5_^3^ | R8_5_^4^ | R12_4_^6^ | R14_4_^7^ | R16_5_^8^ | R18_5_^9^ | ***R10_6_^5^*** | ***R14_6_^7^*** | ***R6_7_^3^*** |
| ***R8_7_^4^*** | ***R10_7_^5^*** | ***R12_7_^6^*** | ***R16_7_^8^*** | ***R18_7_^9^*** | ***R20_7_^10^*** | ***R6_8_^3^*** | ***R10_8_^5^*** | ***R14_8_^7^*** | ***R18_8_^9^*** |
| ***R8_9_^4^*** | ***R10_9_^5^*** | ***R14_9_^7^*** | ***R16_9_^8^*** | ***R20_9_^10^*** | ***R6_10_^3^*** | ***R14_10_^7^*** | ***R18_10_^9^*** |  |  |
