# Supplementary material for: Dipolar Order Parameters in Large Systems With Fast Spinning
Source: Front Mol Biosci. 2021 Dec 9;8:791026. doi: 10.3389/fmolb.2021.791026 (PMC8699854; doi:10.3389/fmolb.2021.791026)
Supplement: Supplementary file 2 [file DataSheet1.zip › TableS5.docx]

Table S5. The following R-symmetries recouple the Heteronuclear dipole-dipole coupling, scalar coupling, and chemical shift anisotropy on the applied nucleus.

| R-Symmetries for Heteronuclear dipole-dipole coupling with γ-encoding | | | | | | | | | | | | | | | | | |
| --- | --- | --- | --- | --- | --- | --- | --- | --- | --- | --- | --- | --- | --- | --- | --- | --- | --- |
| 10 ≤ N ≤ 42; 3 ≤ n ≤ 25; 0.100 kHz ≤ B1 ≤150 kHz; SW ≥ 500 Hz | | | | | | | | | | | | | | | | | |
| N | n | ν | N | n | ν | N | n | ν | N | n | ν | N | n | ν | N | n | ν |
| 10 | 3 | 1 | 14 | 23 | 3 | 20 | 19 | 8 | 26 | 10 | 7 | 32 | 9 | 2 | 38 | 10 | 1 |
|  | 7 | 1 |  | 24 | 1 |  | 21 | 8 |  | 11 | 9 |  | 11 | 6 |  | 11 | 3 |
|  | 9 | 3 |  | 25 | 1 |  | 23 | 4 |  | 12 | 11 |  | 13 | 10 |  | 12 | 5 |
|  | 11 | 3 | 16 | 5 | 2 | 22 | 5 | 1 |  | 14 | 11 |  | 15 | 14 |  | 13 | 7 |
|  | 13 | 1 |  | 7 | 6 |  | 6 | 1 |  | 15 | 9 |  | 17 | 14 |  | 14 | 9 |
|  | 17 | 1 |  | 9 | 6 |  | 7 | 3 |  | 16 | 7 |  | 19 | 10 |  | 15 | 11 |
|  | 19 | 3 |  | 11 | 2 |  | 8 | 5 |  | 17 | 5 |  | 21 | 6 |  | 16 | 13 |
|  | 21 | 3 |  | 13 | 2 |  | 9 | 7 |  | 18 | 3 |  | 23 | 2 |  | 17 | 15 |
|  | 23 | 1 |  | 15 | 6 |  | 10 | 9 |  | 19 | 1 |  | 25 | 2 |  | 18 | 17 |
| 12 | 5 | 4 |  | 17 | 6 |  | 12 | 9 |  | 20 | 1 | 34 | 7 | 3 |  | 20 | 17 |
|  | 7 | 4 |  | 19 | 2 |  | 13 | 7 |  | 21 | 3 |  | 8 | 1 |  | 21 | 15 |
|  | 11 | 4 |  | 21 | 2 |  | 14 | 5 |  | 22 | 5 |  | 9 | 1 |  | 22 | 13 |
|  | 13 | 4 |  | 23 | 6 |  | 15 | 3 |  | 23 | 7 |  | 10 | 3 |  | 23 | 11 |
|  | 17 | 4 |  | 25 | 6 |  | 16 | 1 |  | 24 | 9 |  | 11 | 5 |  | 24 | 9 |
|  | 19 | 4 | 18 | 4 | 1 |  | 17 | 1 |  | 25 | 11 |  | 12 | 7 |  | 25 | 7 |
|  | 23 | 4 |  | 5 | 1 |  | 18 | 3 | 28 | 9 | 4 |  | 13 | 9 | 40 | 9 | 2 |
|  | 25 | 4 |  | 7 | 5 |  | 19 | 5 |  | 11 | 8 |  | 14 | 11 |  | 11 | 2 |
| 14 | 3 | 1 |  | 8 | 7 |  | 20 | 7 |  | 13 | 12 |  | 15 | 13 |  | 13 | 6 |
|  | 4 | 1 |  | 10 | 7 |  | 21 | 9 |  | 15 | 12 |  | 16 | 15 |  | 17 | 14 |
|  | 5 | 1 |  | 11 | 5 |  | 23 | 9 |  | 17 | 8 |  | 18 | 15 |  | 19 | 18 |
|  | 6 | 5 |  | 13 | 1 |  | 24 | 7 |  | 19 | 4 |  | 19 | 13 |  | 21 | 18 |
|  | 8 | 5 |  | 14 | 1 |  | 25 | 5 |  | 23 | 4 |  | 20 | 11 |  | 23 | 14 |
|  | 9 | 3 |  | 16 | 1 | 24 | 5 | 2 |  | 25 | 8 |  | 21 | 9 | 42 | 10 | 1 |
|  | 10 | 1 |  | 17 | 7 |  | 7 | 2 | 30 | 7 | 1 |  | 22 | 7 |  | 11 | 1 |
|  | 11 | 1 |  | 19 | 7 |  | 11 | 10 |  | 8 | 1 |  | 23 | 5 |  | 13 | 5 |
|  | 12 | 3 |  | 20 | 5 |  | 13 | 10 |  | 11 | 7 |  | 24 | 3 |  | 16 | 11 |
|  | 13 | 5 |  | 22 | 1 |  | 17 | 2 |  | 13 | 11 |  | 25 | 1 |  | 17 | 13 |
|  | 15 | 5 |  | 23 | 1 |  | 19 | 2 |  | 14 | 13 | 36 | 11 | 4 |  | 19 | 17 |
|  | 16 | 3 |  | 25 | 5 |  | 23 | 10 |  | 16 | 13 |  | 13 | 8 |  | 20 | 19 |
|  | 17 | 1 | 20 | 7 | 4 |  | 25 | 10 |  | 17 | 11 |  | 17 | 16 |  | 22 | 19 |
|  | 18 | 1 |  | 9 | 8 | 26 | 6 | 1 |  | 19 | 7 |  | 19 | 16 |  | 23 | 17 |
|  | 19 | 3 |  | 11 | 8 |  | 7 | 1 |  | 22 | 1 |  | 23 | 8 |  | 25 | 13 |
|  | 20 | 5 |  | 13 | 4 |  | 8 | 3 |  | 23 | 1 |  | 25 | 4 |  |  |  |
|  | 22 | 5 |  | 17 | 4 |  | 9 | 5 | 32 | 7 | 2 | 38 | 9 | 1 |  |  |  |
