# Supplementary material for: Dipolar Order Parameters in Large Systems With Fast Spinning
Source: Front Mol Biosci. 2021 Dec 9;8:791026. doi: 10.3389/fmolb.2021.791026 (PMC8699854; doi:10.3389/fmolb.2021.791026)
Supplement: Supplementary file 2 [file DataSheet1.zip › TableS6.docx]

Table S6. The following variable flip angle R-Symmetry elements were tested in Simpson. . Red arrows represent the first portion of the composite pulse τ_1_. τ_2_ is the second portion, in blue. τ_3_ is the third portion in yellow. τ_4_ is the third portion in black. τ_5_ is the fifth portion in gray.

| R-element |  |  | k_p_ (B_1_=k_p_ω_r_N/2n) | τ_1,2,3…_ •­ (2τ_r_n/N) |
| --- | --- | --- | --- | --- |
| θ_(0)_ [180+θ]_(180)_ | 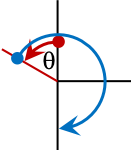 | 1a | (180+2θ)/180 | τ_1_= θ/(180+2θ)  τ_2_= (180+θ)/(180+2θ) |
| θ_(0)_ [180+2θ]_(180)_ θ_(0)_ | 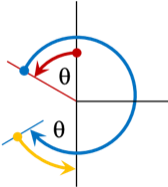 | 2a | (180+4θ)/180 | τ_1_= θ/(180+4θ)  τ_2_= (180+2θ)/(180+4θ)  τ_3_= θ/(180+4θ) |
| [180+2θ]_(0)_ 3θ_(180)_ θ_(0)_ | 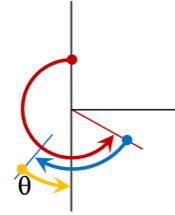 | 2b | (180+6θ)/180 | τ_1_= (180+2θ)/(180+6θ)  τ_2_= (3θ)/(180+6θ)  τ_3_= (θ)/(180+6θ) |
| [90+θ]_(0)_ 2θ_(180)_ [90+θ]_(0)_ | 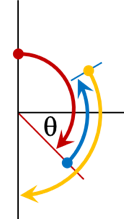 | 2c | (180+4θ)/180 | τ_1_= (90+θ)/(180+4θ)  τ_2_= (2θ)/(180+4θ)  τ_3_= (90+θ)/(180+4θ) |
| θ_(0)_ θ_(180)_ 180_(0)_ | 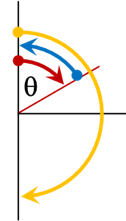 | 2d | (180+2θ)/180 | τ_1_= (θ)/(180+2θ)  τ_2_= (θ)/(180+2θ)  τ_3_= (180)/(180+2θ) |
| θ_(0)_ 2θ_(180)_ [180+θ]_(0)_ | 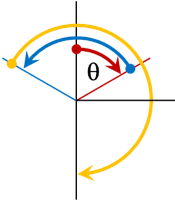 | 2e | (180+4θ)/180 | τ_1_= (θ)/(180+4θ)  τ_2_= (2θ)/(180+4θ)  τ_3_= (180+θ)/(180+4θ) |
| θ_(0)_ [180+2θ]_(180)_ [360+θ]_(0)_ | 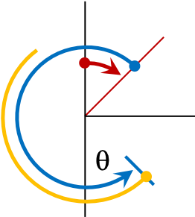 | 2f | (540+4θ)/180 | τ_1_= (θ)/(540+4θ)  τ_2_= (180+2θ)/(540+4θ)  τ_3_= (360+θ)/(540+4θ) |
| θ_(0)_ [180+2θ]_(180)_ 2θ_(0)_ θ_(180)_ | 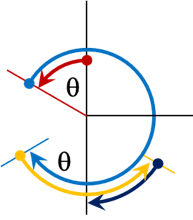 | 3a | (180+6θ)/180 | τ_1_= (θ)/(180+6θ)  τ_2_= (180+2θ)/(180+6θ)  τ_3_= (2θ)/(180+6θ)  τ_4_= (θ)/(180+6θ) |
| [90+θ]_(0)_ 2θ_(180)_ [90+2θ]_(0)_ θ_(180)_ | 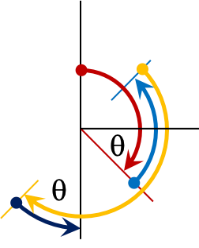 | 3b | (180+6θ)/180 | τ_1_= (90+θ)/(180+6θ)  τ_2_= (2θ)/(180+6θ)  τ_3_= (90+2θ)/(180+6θ)  τ_4_= (θ)/(180+6θ) |
| θ_(0)_ θ_(180)_ [180+θ]_(0)_ θ_(180)_ | 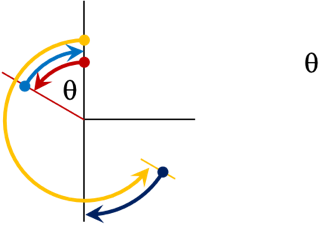 | 3c | (180+4θ)/180 | τ_1_= (θ)/(180+4θ)  τ_2_= (θ)/(180+4θ)  τ_3_= (180+θ)/(180+4θ)  τ_4_= (θ)/(180+4θ) |
| 90_(0)_ θ_(90)_ 90_(0)_ | 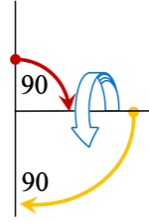 | 4a | (180+θ)/180 | τ_1_= (90)/(180+θ)  τ_2_= (θ)/(180+θ)  τ_3_= (90)/(180+θ) |
| θ_(0)_ 180_(90)_ θ_(0)_ | 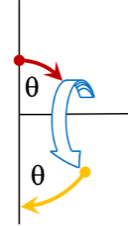 | 4b | (180+2θ)/180 | τ_1_= (θ)/(180+2θ)  τ_2_= (180)/(180+2θ)  τ_3_= (θ)/(180+2θ) |
| θ_(0)_ 360_(90)_ [180+θ]_(0)_ | 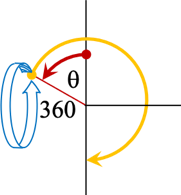 | 5a | (540+6θ)/180 | τ_1_= (θ)/(540+2θ)  τ_2_= (360)/(540+2θ)  τ_3_= (180+θ)/(540+2θ) |
| 90_(0)_ θ_(90)_ 360_(0)_ | 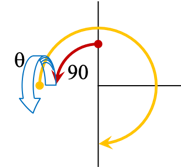 | 5b | (360+θ)/180 | τ_1_= (90)/(360+θ)  τ_2_= (θ)/(360+θ)  τ_3_= (270)/(360+θ) |
| 90_(0)_ θ_(90)_ θ_(270)_ 90_(0)_ | 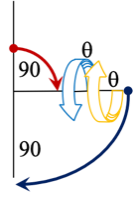 | 6a | (180+2θ)/180 | τ_1_= (90)/(180+2θ)  τ_2_= (θ)/(180+2θ)  τ_3_= (θ)/(180+2θ)  τ_4_= (90)/(180+2θ) |
| θ_(0)_ 90_(90)_ 270_(270)_ θ_(0)_ | 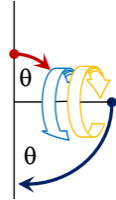 | 6b | (360+2θ)/180 | τ_1_= (θ)/(360+2θ)  τ_2_= (90)/(360+2θ)  τ_3_= (270)/(360+2θ)  τ_4_= (θ)/(360+2θ) |
| 90_(0)_ θ_(90)_ θ_(270)_ 270_(0)_ | 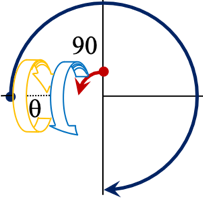 | 7a | (360+6θ)/180 | τ_1_= (90)/(360+2θ)  τ_2_= (θ)/(360+2θ)  τ_3_= (θ)/(360+2θ)  τ_4_= (270)/(360+2θ) |
| θ_(0)_ 2θ_(180)_ [180+2θ]_(0)_ 2θ_(180)_θ_(0)_ | 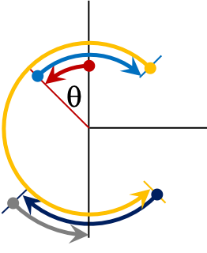 | 8a | (180+8θ)/180 | τ_1_= (θ)/(180+8θ)  τ_2_= (2θ)/(180+8θ)  τ_3_= (180+2θ)/(180+8θ)  τ_4_= (2θ)/(180+8θ)  τ_5_= (θ)/(180+8θ) |
| [90+θ]_(0)_ 2θ_(180)_ [180+2θ]_(0)_ 2θ_(180)_θ_(0)_ | 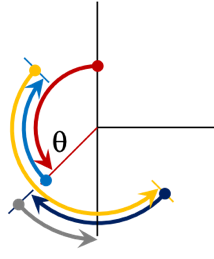 | 8b | (180+8θ)/180 | τ_1_= (90+θ)/(180+8θ)  τ_2_= (2θ)/(180+8θ)  τ_3_= (90+2θ)/(180+8θ)  τ_4_= (2θ)/(180+8θ)  τ_5_= (θ)/(180+8θ) |
| θ_(0)_ [90+2θ]_(180)_ [180+2θ]_(0)_ 2θ_(180)_θ_(0)_ | 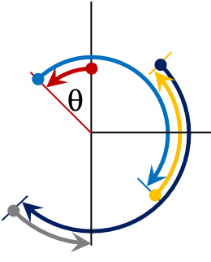 | 8c | (180+8θ)/180 | τ_1_= (θ)/(180+8θ)  τ_2_= (90+2θ)/(180+8θ)  τ_3_= (2θ)/(180+8θ)  τ_4_= (90+2θ)/(180+8θ)  τ_5_= (θ)/(180+8θ) |
| θ_(0)_ θ_(180)_ [180+θ]_(0)_ 2θ_(180)_ θ_(0)_ | 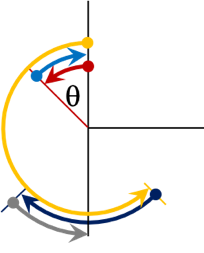 | 8d | (180+6θ)/180 | τ_1_= (θ)/(180+6θ)  τ_2_= (θ)/(180+6θ)  τ_3_= (180+θ)/(180+6θ)  τ_4_= (2θ)/(180+6θ)  τ_5_= (θ)/(180+6θ) |
| [180+θ]_(0)_ θ_(180)_ θ_(0)_ 2θ_(180)_ θ_(0)_ | 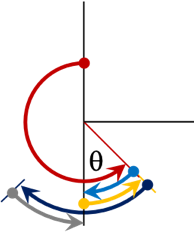 | 8e | (180+6θ)/180 | τ_1_= (180+θ)/(180+6θ)  τ_2_= (θ)/(180+6θ)  τ_3_= (θ)/(180+6θ)  τ_4_= (2θ)/(180+6θ)  τ_5_= (θ)/(180+6θ) |
