# Supplementary figures and images for: Dipolar Order Parameters in Large Systems With Fast Spinning
Source: Front Mol Biosci. 2021 Dec 9;8:791026. doi: 10.3389/fmolb.2021.791026 (PMC8699854; doi:10.3389/fmolb.2021.791026)

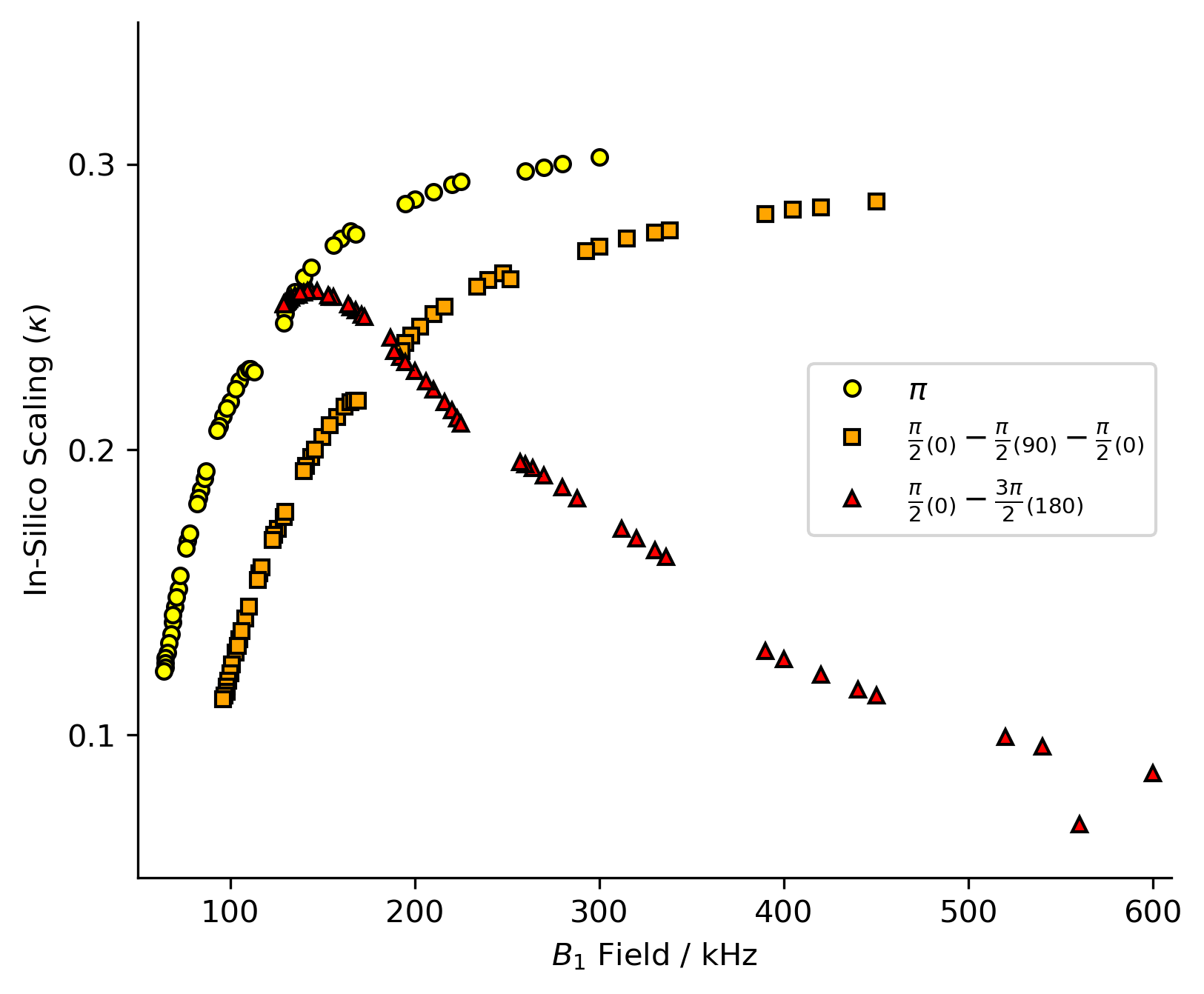

Supplement: Supplementary file 2 [file DataSheet1.zip › FigS1.png]

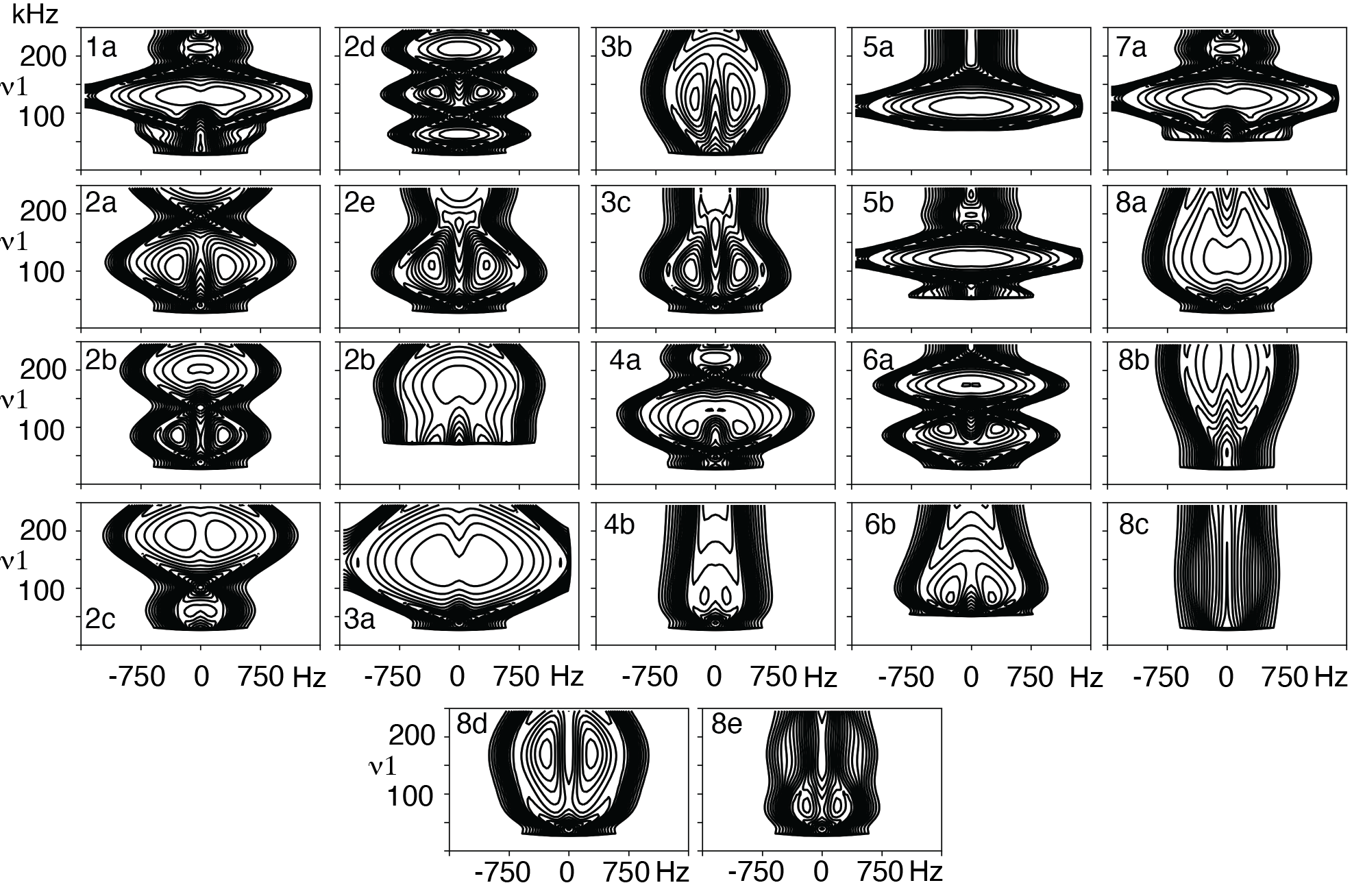

Supplement: Supplementary file 2 [file DataSheet1.zip › FigS2.png]

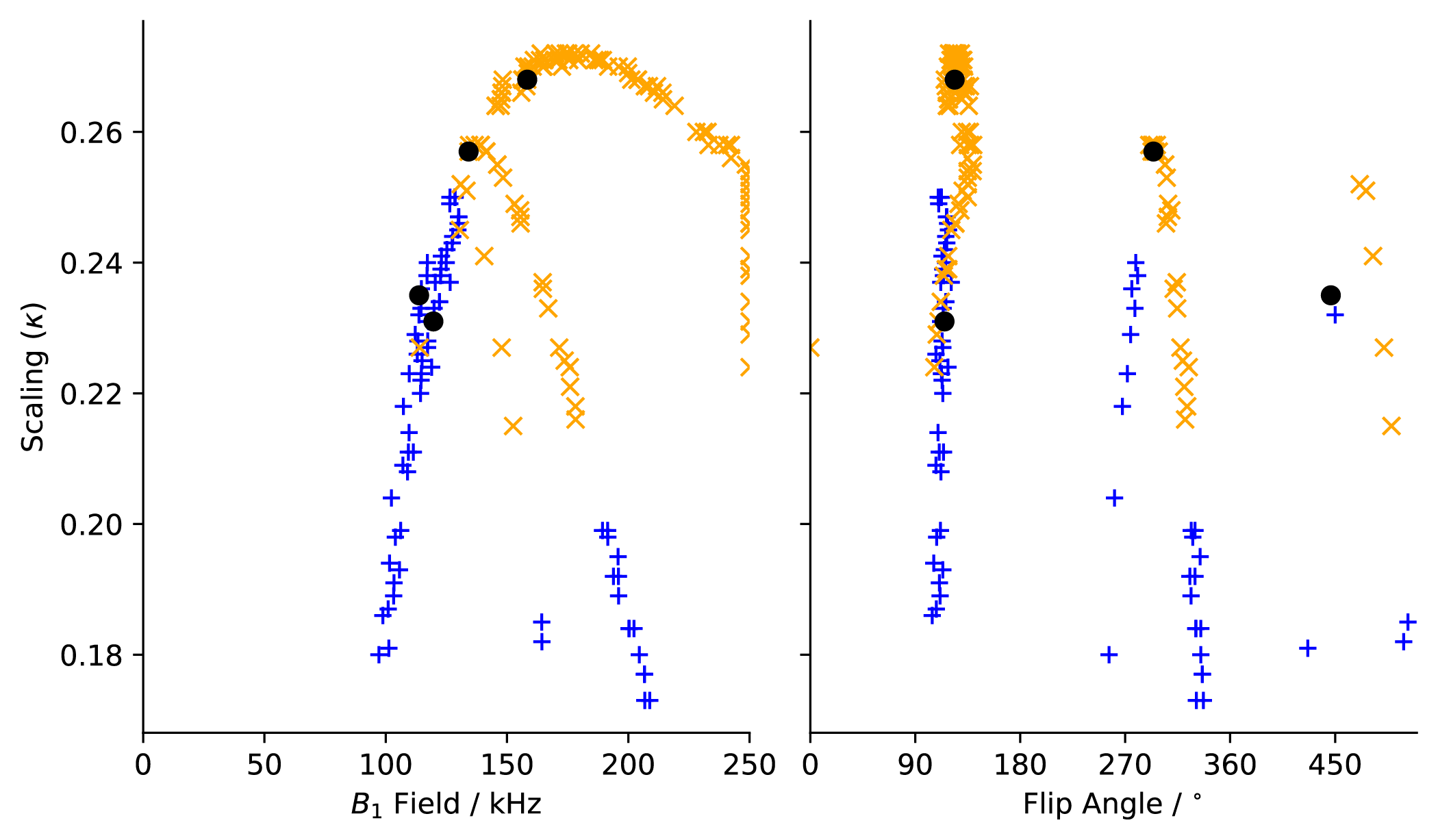

Supplement: Supplementary file 2 [file DataSheet1.zip › FigS3.png]

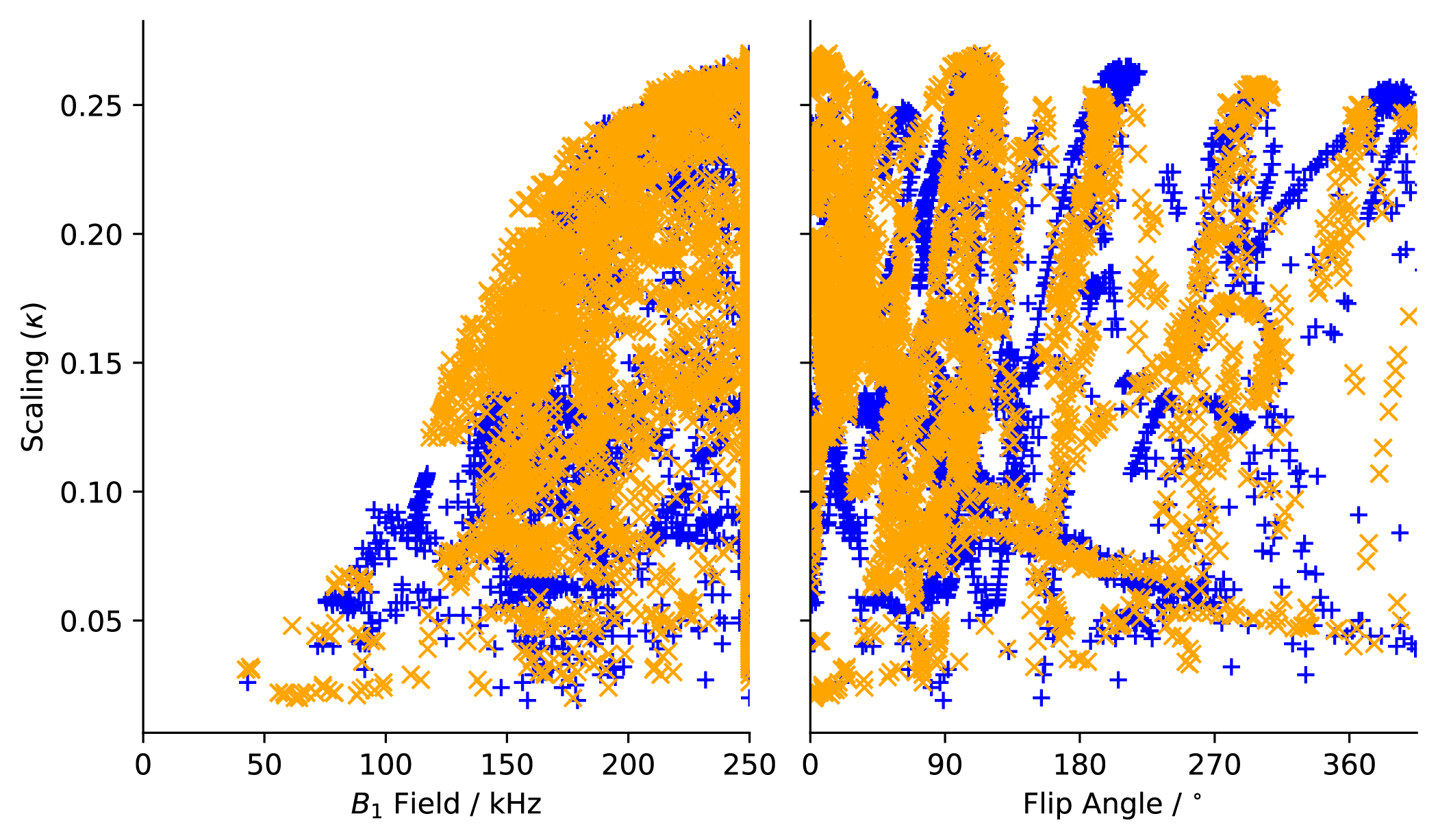

Supplement: Supplementary file 2 [file DataSheet1.zip › FigS4.png]

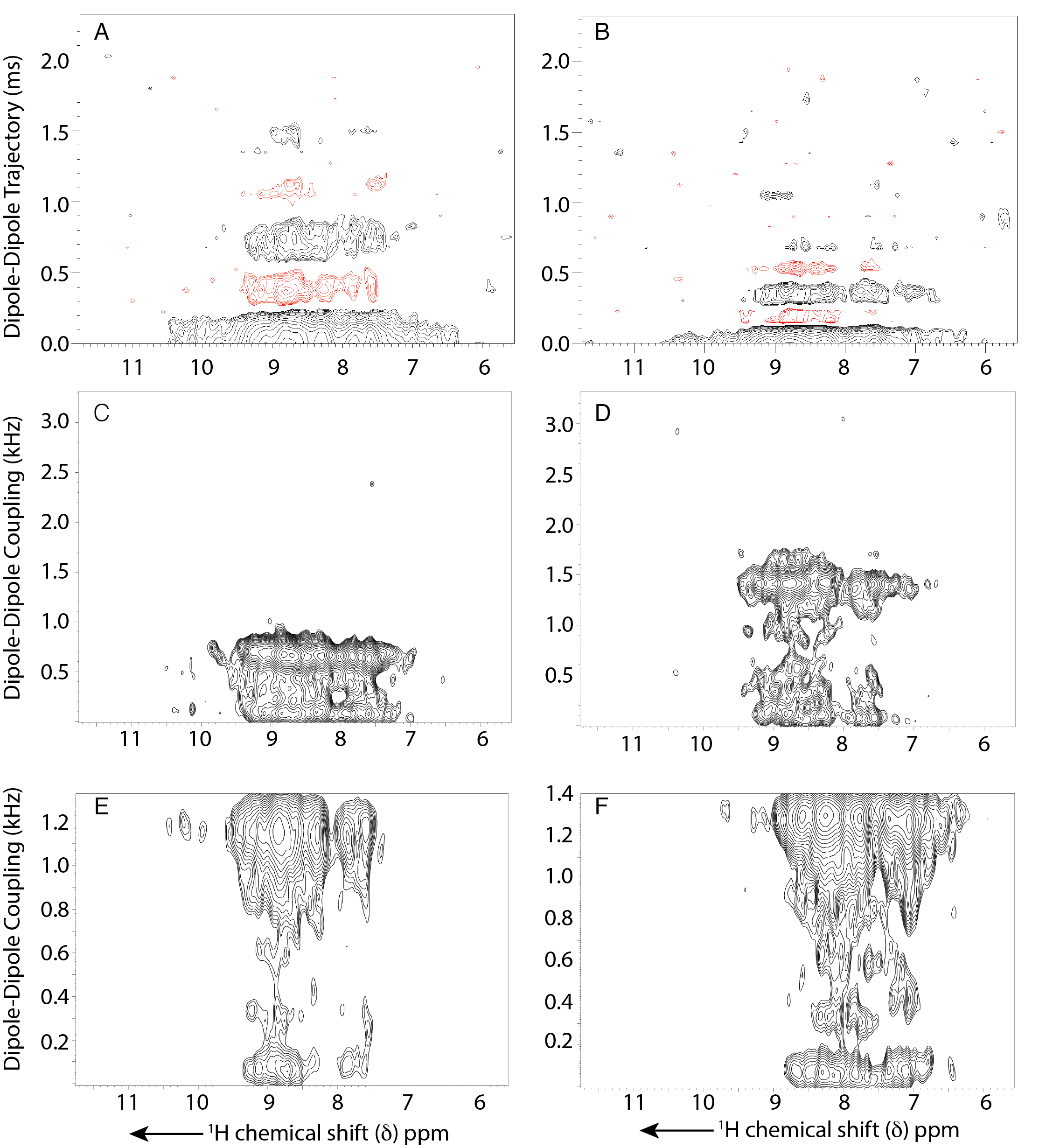

Supplement: Supplementary file 2 [file DataSheet1.zip › FigS5.png]

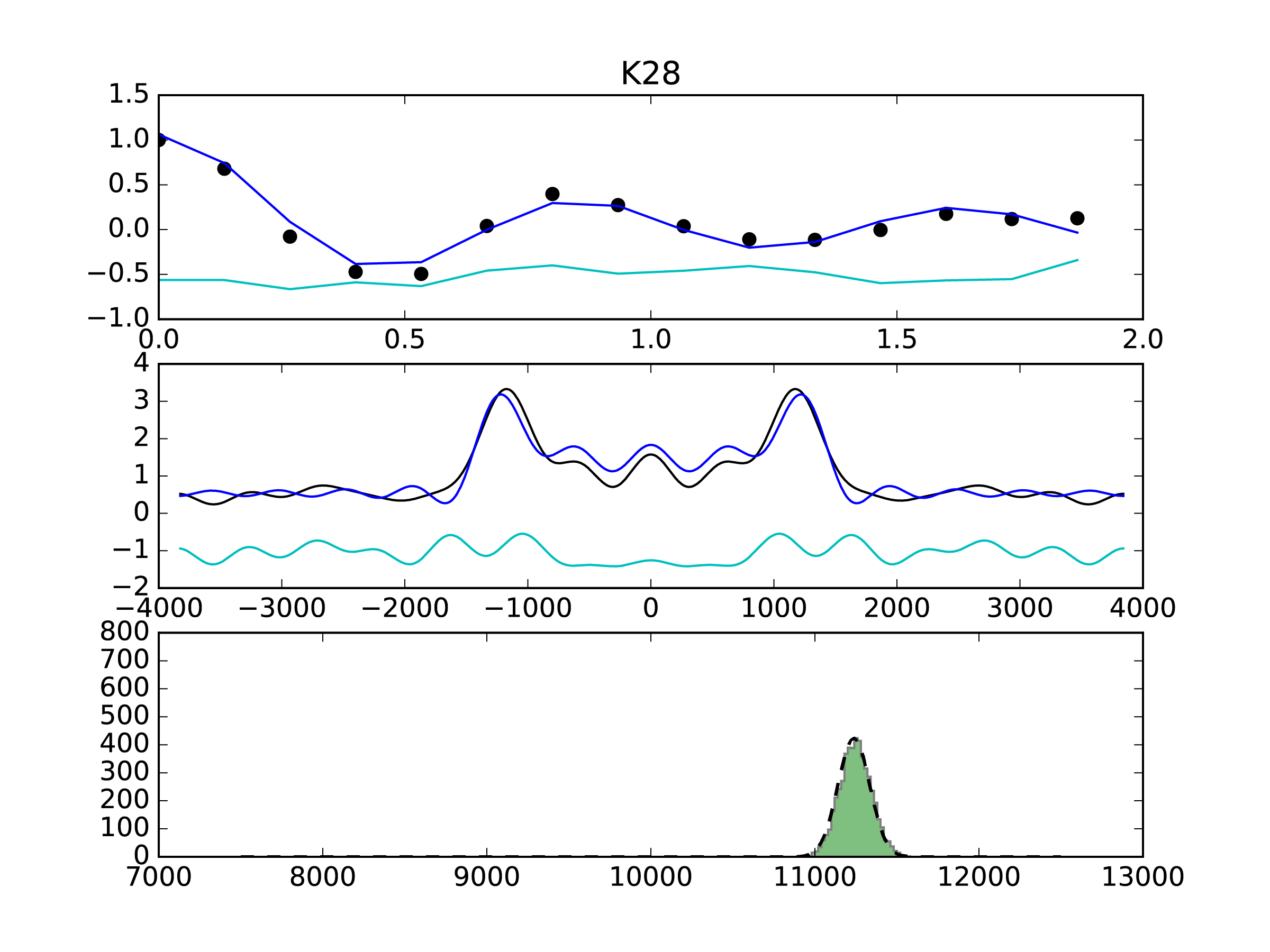

Supplement: Supplementary file 2 [file DataSheet1.zip › FigS6.png]

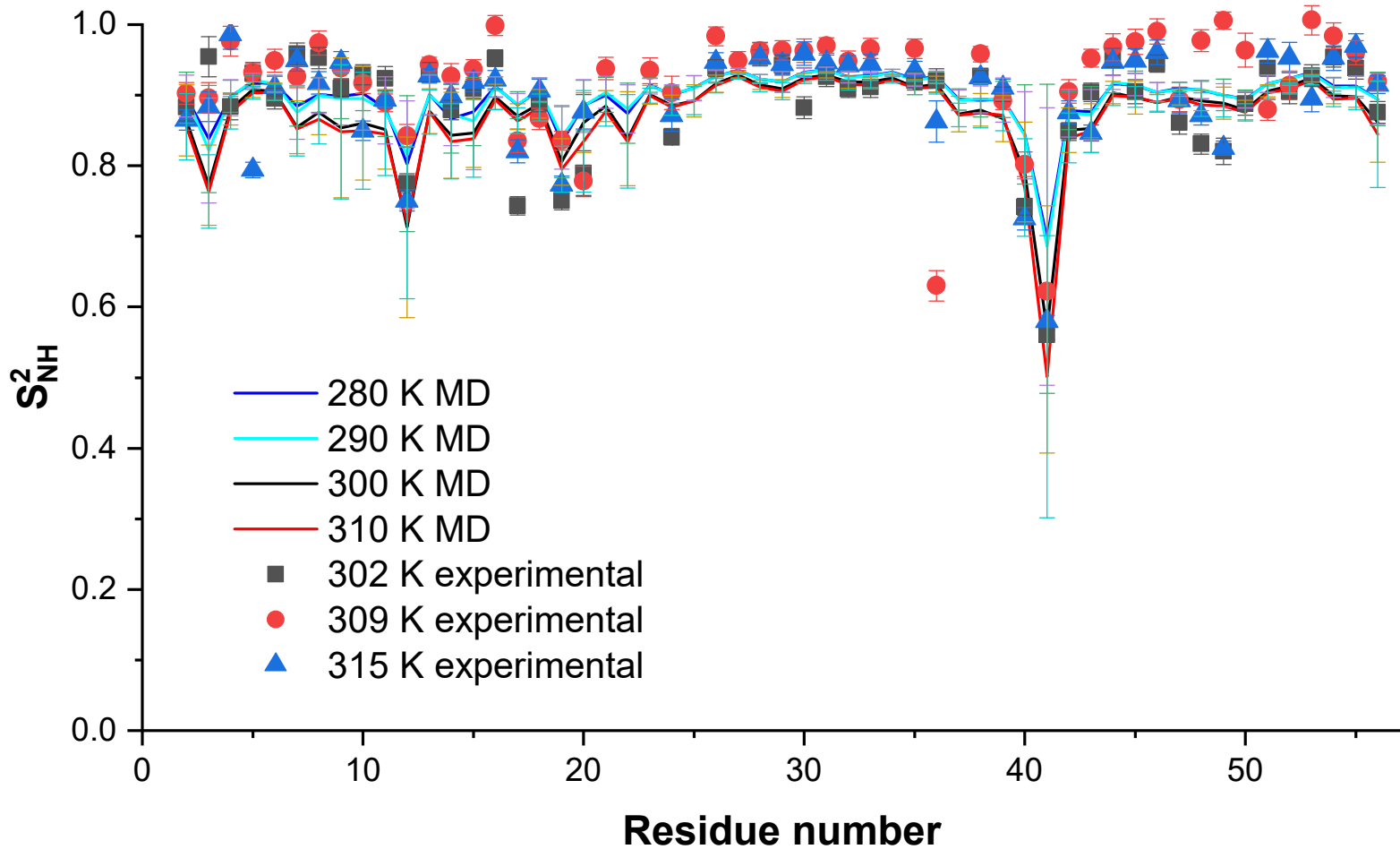

Supplement: Supplementary file 2 [file DataSheet1.zip › FigS7.pdf]

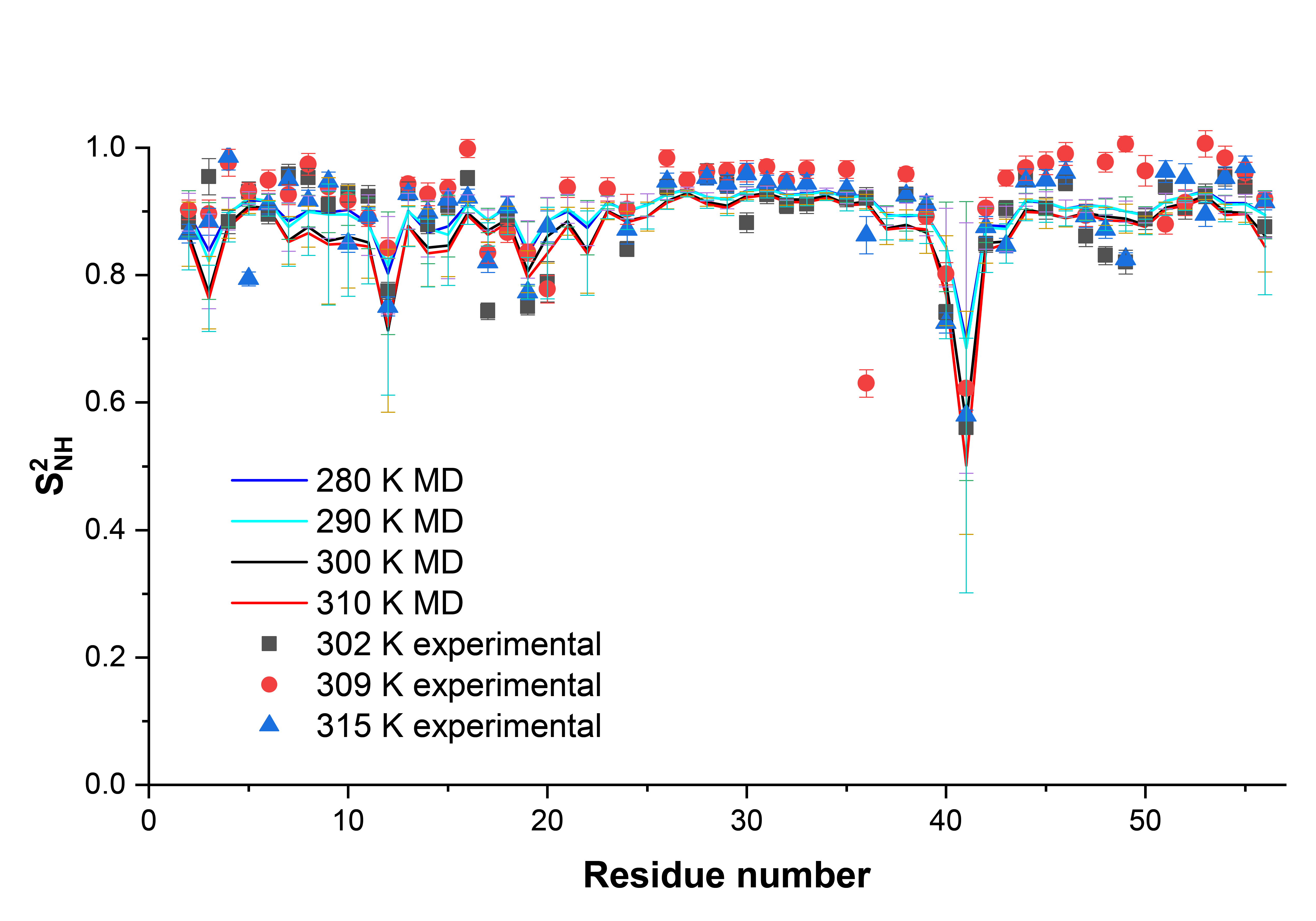

Supplement: Supplementary file 2 [file DataSheet1.zip › FigS7.png]

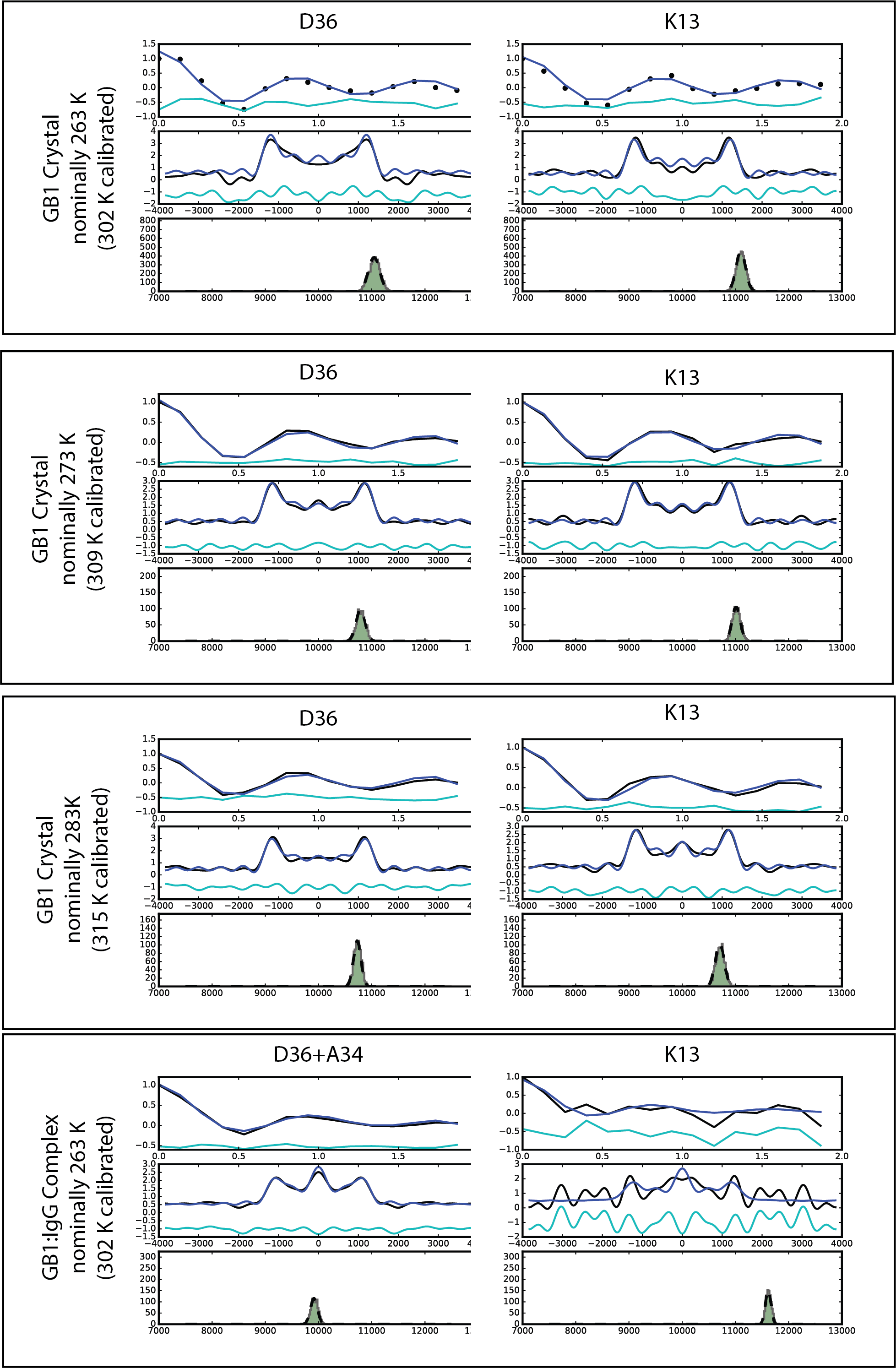

Supplement: Supplementary file 2 [file DataSheet1.zip › FigS8.png]

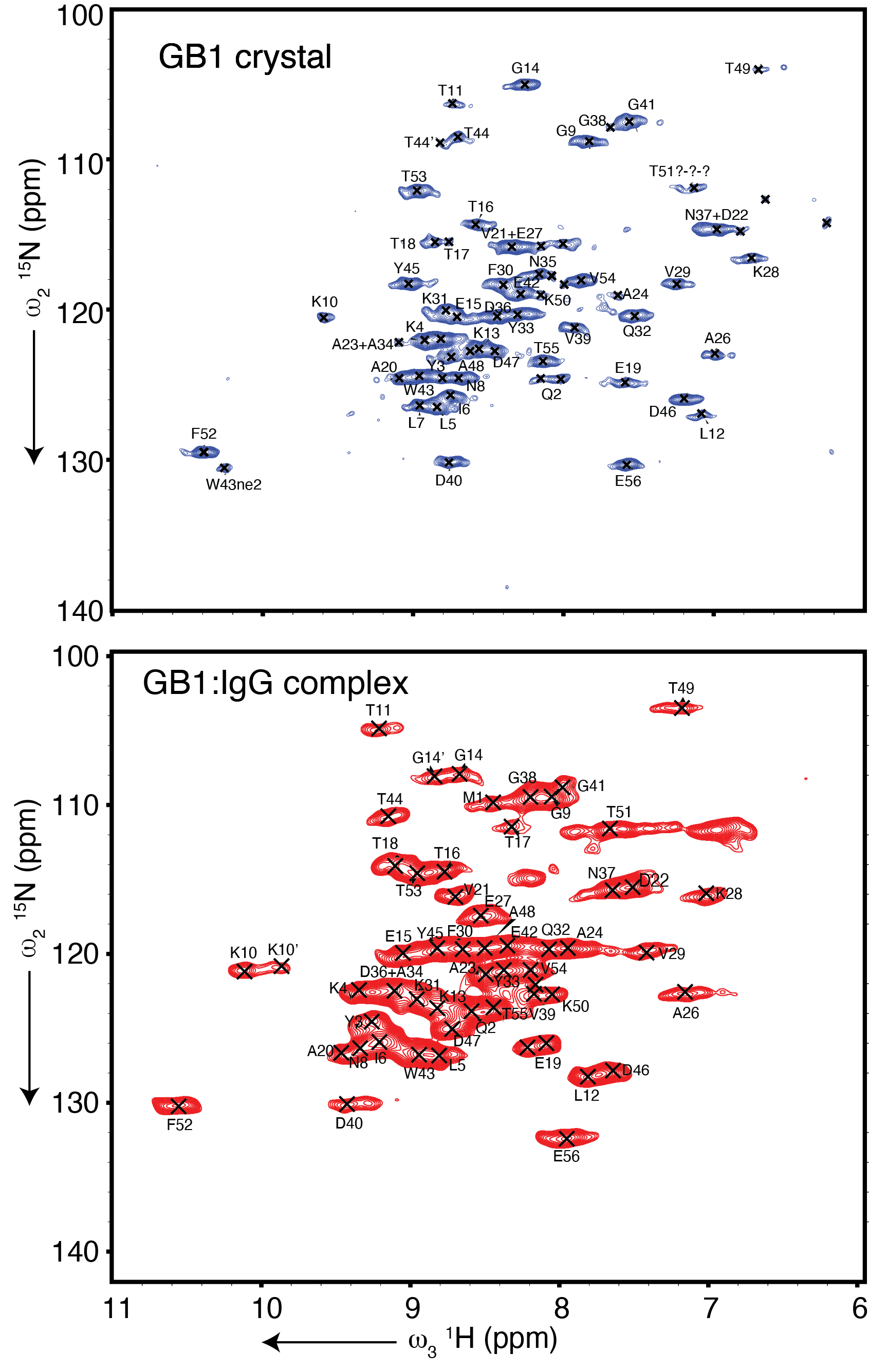

Supplement: Supplementary file 2 [file DataSheet1.zip › FigS9.png]
